# A cross-country comparison of temporal change in adolescent mental health problems in the UK and Brazil

**DOI:** 10.1017/S2045796025000137

**Published:** 2025-03-12

**Authors:** J. M. Armitage, E. Viegas da Silva, F. Tseliou, L. Riglin, G. Hammerton, S. Collishaw, I. S. Santos, L. Tovo-Rodrigues, A. M. B. Menezes, C. F. Wehrmeister, H. Gonçalves, A. Matijasevich, J. Murray

**Affiliations:** 1 Wolfson Centre for Young People’s Mental Health, Cardiff University, Wales, UK; 2 Division of Psychological Medicine and Clinical Neurosciences, Centre for Neuropsychiatric Genetics and Genomics, Cardiff University, Wales, UK; 3 Postgraduate Program in Epidemiology, Federal University of Pelotas, Pelotas, Brazil; 4 Human Development and Violence Research Centre (DOVE), Federal University of Pelotas, Pelotas, Brazil; 5 State Health Surveillance Centre of Rio Grande do Sul, Porto Alegre, Brazil; 6 Centre for Academic Mental Health, Population Health Sciences, Bristol Medical School, University of Bristol, Bristol, UK; 7 Medical Research Council Integrative Epidemiology Unit at the University of Bristol, Population Health Sciences, Bristol Medical School, University of Bristol, Bristol, UK; 8 Department of Preventive Medicine, Faculty of Medicine of University of São Paulo (FMUSP), University of São Paulo, São Paulo, Brazil

**Keywords:** adolescence, cross-country, mental health, trends, cohort, sdq, population, ALSPAC, MCS, Pelotas

## Abstract

**Aims:**

Epidemiological evidence shows a concerning rise in youth mental health difficulties over the past three decades. Most evidence, however, comes from countries in Europe or North America, with far less known about changes in other global regions. This study aimed to compare adolescent mental health across two population-based cohorts in the UK, and two population-based cohorts in Pelotas, Brazil.

**Methods:**

Four population-based cohorts with identical mental health measures were compared. In Brazil, these included the 1993 Pelotas Birth Cohort and the 2004 Pelotas Birth Cohort. In the UK, cohorts included the Avon Longitudinal Study of Parents and Children, and the Millennium Cohort Study. Mental health was measured in all cohorts using identical, parent-rated scores from the Strengths and Difficulties Questionnaire (SDQ). This was assessed in both countries over approximately the same time periods, when adolescents were aged 11 (2004 vs 2015 in Brazil, and 2003 vs 2012 in the UK), with follow-up analyses focused on outcomes in later adolescence.

**Results:**

Mental health problems were higher in the UK for adolescents born in the early 2000s compared to those born in the early 1990s. In Pelotas, the opposite was found, whereby problems were lower for adolescents born in the early 2000s compared to those born in the early 1990s. Despite these promising reductions in mental health problems in Pelotas over time, SDQ scores remained higher in Pelotas compared to the UK.

**Conclusions:**

Our study represents the first to compare two population-based cohorts in the UK, and two population-based cohorts in Pelotas, Brazil, to understand how mental health problems have changed over time across the two settings. Our findings provide the most up-to-date insight into population-level rates of youth mental health problems in Pelotas, and shed novel insight into how these have changed over the last two decades in comparison to the UK. In doing so, our study provides a tentative first step towards understanding youth mental health over time at a more global scale, and presents a valuable opportunity to examine putative contributors to differences across time.

## Introduction

Mental health problems affect 10–20% of children and adolescents worldwide and stand as a leading cause of the global burden of disease (Baranne and Falissard, 2018). Mental health problems are distressing for young people and their families, with both immediate and long-term consequences for psychosocial development and health (Thapar *et al.*, 2022). Epidemiological evidence from high-income countries has demonstrated increasing rates of youth mental health difficulties, particularly emotional problems, over the past few decades (Armitage *et al.*, 2023a; Collishaw and Sellers, 2020). Yet global data are severely limited, with most studies based in select countries in Europe and North America. Although approaches to tackling mental health problems will vary by country, examining whether increasing trends are global will help to understand the magnitude of the issue and could allude to possible local versus global mechanisms. Comparing across settings could thus be crucial to identifying environmental factors driving population-level.

In Brazil, child and adolescent mental health is a prominent public health concern (Mari, 2014). Brazil differs in important respects to the UK and other countries in which increased mental health problems are documented. Brazil has witnessed rapid demographic, economic, nutritional and educational changes over the last few decades (Bertoldi *et al.*, 2019). In combination with existing inequalities and other social risks like poverty and urbanisation, these changes are likely to have substantial and varying influences on trends in mental health across time. Some studies have compared youth mental health problems over time in Brazil, but most are limited to children under 5 years (Degli *et al.*, 2023; Matijasevich *et al.*, 2014). Investigating change among older children to test how changes compare to the UK represents an important opportunity to understand more about cross-country differences.

The current study compares youth mental health across two population-based cohorts in Pelotas, Brazil, and two population-based cohorts in the UK. The aim was to first understand how youth mental health problems have changed over the last two decades in each location, and secondly how changes compare between them. Previous research in the UK suggests that increases in mental health problems have been particularly pronounced for emotional problems amongst female adolescents.^3^ In Brazil, increases over time have been documented for emotional and behavioural problems among children, but no clear sex differences have been found (Degli *et al.*, 2023). This is likely due to having limited analyses to younger children, as sex differences typically emerge later in development (Armitage *et al.*, 2023a). The current study therefore examined, for the first time, mental health changes among male and female adolescents in Pelotas. We investigate total mental health difficulties, as well as individual subscales capturing emotional, conduct, hyperactivity and peer problems. It was predicted that there would be increases in youth mental health problems over time in both Pelotas, Brazil and in the UK, and these would be greatest for older adolescents. It was also predicted that rates would be higher across all ages and time points in Pelotas given higher rates of inequality and social risks present in Brazil.

## Methods

### Samples and study design

Four large, population-based cohorts covering the first two decades of the 21st century were used to compare rates of change in adolescent mental health. In the UK, we use the Avon Longitudinal Study of Parents and Children (ALSPAC) and the Millennium Cohort Study (MCS). In Brazil, two cohorts from Pelotas were used (1993 Pelotas Birth Cohort and 2004 Pelotas Birth Cohort). Main analyses focus on mental health at 11 years of age, with follow-up analyses focused on change in adolescent mental health.

### Avon Longitudinal Study of Parents and Children

The Avon Longitudinal Study of Parents and Children (ALSPAC’91) is a birth cohort that recruited pregnant women residing in the former Avon area in the South West of the UK, with expected delivery dates between 1 April 1991, and 31 December 1992 (Boyd *et al.*, 2013). The number of pregnancies enrolled was 14,541, with 13,988 children alive at age 1 year (Fraser *et al.*, 2013). Recruitment was opportunistic and achieved through the media and at routine antenatal and maternity health services. When the children were approximately 7 years, an attempt was made to bolster the sample with eligible families who had failed to join the study originally, increasing the sample to 15,447 pregnancies and 14,901 children alive at 1 year. Both parents and children have been followed up regularly since recruitment. The current study uses assessments that took place in 2002–2003 when participants were aged 11, and in 2008–2009 when participants were approximately 17 years. The study website contains details of all the data that is available through a fully searchable data dictionary and variable search tool (http://www.bristol.ac.uk/alspac/researchers/our-data/).

Ethical approval for the ALSPAC study was obtained from ALSPAC Ethics and Law Committee and the Local Research Ethics Committees. Informed consent for the use of data collected via questionnaires and clinics was obtained from participants following the recommendations of the ALSPAC Ethics and Law Committee at the time.

### Millennium Cohort Study

The Millennium Cohort Study (MCS’00) is a longitudinal study of 18,552 families (18,827 children) born between 1 September 2000 and 11 January 2002, in England (63.6%), Wales (14.3%), Scotland (12.1%) or Northern Ireland (10.0%) (Connelly *et al.*, 2014). Eligible children were identified using government child benefit records, a benefit with almost universal coverage at that time. At age 3, a total of 692 new eligible families were recruited, bringing the total number of children to 19,517 (19,243 families). A key asset of the recruitment process was that efforts were made to ensure adequate representation of diverse communities across the four UK countries through oversampling (Plewis, 2007). To account for this selection process, sample designs weights were used in the present analyses (see www.cls.ioe.ac.uk). Participants have been assessed across seven sweeps, with this study using assessments in 2012 when participants were aged 11, and in 2018, when participants were 17 years. Ethical approval for the MCS was obtained by the London Multi‐Centre Research Ethics Committee.

### 1993 Pelotas birth cohort

Pelotas is a city located in the South of Brazil and in 2022 had an estimated population of 325,685 inhabitants, 93% of whom live in the urban area. The 1993 Pelotas Birth Cohort is a population-based study that recruited babies born between January 1 and 31 December 1993. Eligible participants born during this timeframe were recruited through daily visits to all five hospitals in the city of Pelotas that year. In total, just 16 mothers could not be interviewed at baseline or refused to participate in the study, resulting in 5,249 (99.7%) recruited newborns (Victora *et al.*, 2008). Follow-up home visits to subsamples of the cohort took place throughout childhood, with the first attempt made to include all original participants in 2004 when participants were 11 years of age. Further follow-up visits were carried out in homes and the research clinic, including at 15 years of age (Goncalves *et al.*, 2014).

### 2004 Pelotas birth cohort

The 2004 Pelotas Birth Cohort includes infants born throughout the year of 2004 in the city of Pelotas, following similar procedures to the 1993 Pelotas Birth Cohort study, except that all follow-ups aimed to evaluate all participants in the cohort, not just subsamples. Hospitals with maternity wards were visited daily, and all live births were considered eligible for enrolment in the study (Santos *et al.*, 2011). A total of 4,231 newborns were included in the cohort, representing 99.2% of all births in the city during that year. All participants were assessed at birth, and again across childhood. The current study includes participants assessed in 2015 when participants were 11 years of age, as well as those assessed at the 15-year follow up, which occurred between November 2019 and March 2020. Data collection at the research clinic was interrupted during this wave when social distancing measures took place in Brazil due to the COVID-19 pandemic. At that point, 1,949 adolescents and their caregivers had been interviewed (48.5% of the original cohort invited to participate by birth order).

All 1993 and 2004 Pelotas Birth Cohort follow-ups were approved by the Federal University of Pelotas Medical School Research Ethics Committee.

## Measures

### Mental health problems

Commensurate data across all four cohorts are available using the parent-completed, Strengths and Difficulties Questionnaire (SDQ). The SDQ is an internationally recognised screening instrument for child and adolescent emotional and behavioural difficulties, and it has been validated in both the UK (Armitage *et al.*, 2023a; 2023b) and Brazil (Anselmi *et al.*, 2010). The questionnaire includes four 5-item problem subscales (emotional, conduct, hyperactivity, peer problems) that can be combined into a total difficulties score. We focus on the total difficulty score (0–40), as well as individual subscales ranging from 0 to 10 (mean imputation used for those with ≤2 of items missing). In the UK cohorts, SDQ scores are available at 11, 14 and 17 years of age, and in the Pelotas cohorts, data are available at 11 and 15 years of age. Main analyses therefore focus on age 11, with follow-up analyses comparing outcomes for 14–17 year olds (see below).

## Statistical analyses

Differences in total difficulty scores were compared across the two cohorts within each location and over the same time period, for individuals aged 11 (2004 vs 2015 in Brazil, and 2003 vs 2012 in the UK). Analyses assessed differences in mean problem scores between the two cohorts in each country, as well as rates of clinically significant problems (i.e. abnormal range SDQ symptom scores according to SDQ recommendations, see https://www.sdqinfo.org/py/sdqinfo/c0.py). Analyses were repeated for each of the four SDQ subscales. Differences in change of mean scores over time periods across country were then investigated using linear regression with a country × time interaction. This enabled comparison of whether mean differences over the time periods in the UK differ to mean differences over the same time period in Pelotas. All analyses were repeated after stratifying by sex to enable subgroup comparisons of males and females. To test sex differences over the time period within country, a time × sex interaction was used, and to test differences across both country and time, a three-way interaction was used (country × time × sex).

### Follow-up analyses

Analyses were repeated using data on individuals aged 15 in Brazil, and aged 17 in the UK (2008 vs 2019 in Brazil, and 2008 vs 2018 in the UK). This ensured a similar time period was compared across the two countries. Further analyses, however, were also carried out on the UK cohorts using data available at 14 years. This was to ensure any cross-country differences were not a result of using slightly older adolescents in the UK (assessed at 17 years) compared to in Brazil (assessed at 15 years). Thus, analyses also compared mental health over time period for 14-year olds in the UK, with 15-year olds in Brazil (2008 vs 2019 in Brazil, and 2005 vs 2015 in the UK).

#### Sample weights to enhance representativeness and comparability of the cohorts

The Pelotas cohorts included over 99% of the eligible populations sampled in the Pelotas City, and therefore no sampling weights were required. In contrast, the two UK cohorts differed geographically and in their sampling approach. In order to enhance comparability of the two UK cohorts, two weights were used. First, in ALSPAC, weights were generated aiming to represent the UK population at the time of study recruitment, using data from the 1991 Census Household Sample of Anonymised Records for Great Britain. See Supplementary material for more information. Second, for the MCS, a sample design weight was used to correct for the stratified cluster sample design (Plewis, 2007).

#### Attrition at follow-up and non-response weights

Missing data were handled in each of the four cohorts using inverse probability weighting. Individuals with complete mental health data were weighted by the inverse probability of them being a complete case. This was done using variables available for the full cohort assessed in pregnancy or infancy that were associated with missingness (see Supplementary Table 2 for variables included, and Supplementary Tables 3 for a comparison of weighted and unweighted estimates). In the UK cohorts, analysis weights were created by interacting the non-response weights with the sampling weights. All analyses were carried out in Stata (version 17).

#### Measurement invariance

Analyses were tested for measurement invariance across cohorts (within-country) using multiple group confirmatory factor analysis for (a) UK age 11, (b) UK age 17 years, (c) Pelotas age 11 and (d) Pelotas age 15. This was to test whether the meaning of the SDQ was the same across cohorts being compared within each country. The grouping variable was therefore cohort (within the UK ALSPAC and MCS were compared, and within Brazil, the 1993 and 2004 cohorts were compared). We evaluated increasingly stringent types of measurement invariance (i) configural invariance, (ii) metric (“weak”) invariance and (iii) scalar (“strong”) invariance: more information about these models can be found in the Supplementary material. Measurement invariance was tested separately for UK age 11, UK age 17 years, Pelotas age 11 and Pelotas age 15. As a secondary analysis, we also investigated measurement invariance across the two countries to determine whether the balance and meaning of items differed across the two settings. More information about these analyses can be found in the Supplementary material.

## Results

We found evidence of strong measurement invariance for all subscales across time within each country (see Supplementary Tables 7 and 8). Comparisons of measurement invariance across country also provided evidence of measurement invariance (see Supplementary Tables 9 and 10).

### Main analyses

#### Within-country comparisons of mental health at age 11

**Figure 1.**
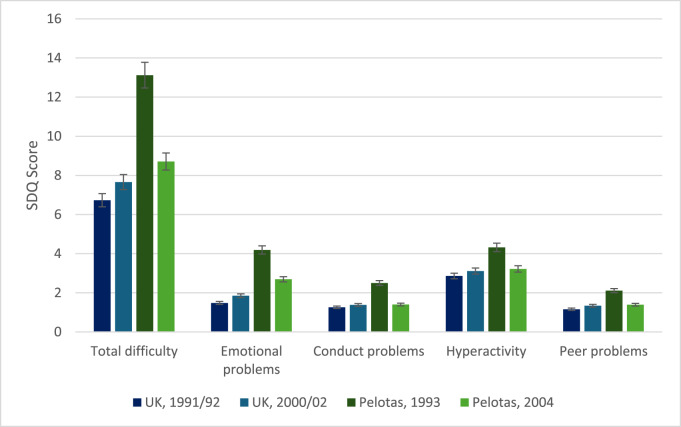
Differences in SDQ total difficulty and subscale scores at age 11 in the UK and Pelotas cohorts. Note total difficulty scores range from 0 to 40, and individual subscales from 0 to 10.

In the UK, total mental health difficulty scores increased for those born in 2000–2002 compared to those born in 1991–1992 (Fig. 1). Specifically, for adolescents born in the early 2000s, mean total difficulty scores were 0.93 (95% CI 0.73, 1.13) higher at age 11 compared to those born in 1991–1992 (Table 1), representing a small overall increase. When comparing the individual subscales among 11-year-olds, increases were noted across the time periods for all four subscales, with increases in abnormal range symptoms greatest for emotional problems (7.3–10.9%).

**Table 1.** Differences in mean and abnormal SDQ scores at 11 years

	UK				Brazil					
	ALSPAC 1991−1992	MCS 2000−2002	Change in UK^a^		Pelotas cohort 1993	Pelotas cohort 2004	Change in Brazil^a^		Difference between changes over time in UK and changes over time in Brazil	
11 years	Mean (95% CI)	Mean (95% CI)	Estimate	*P* value	Mean (95% CI)	Mean (95% CI)	Estimate	*P* value	Difference^a^	*P* value
**Total SDQ**										
Mean	6.73 (6.59, 6.89)	7.66 (7.54, 7.79)	0.93 (0.73, 1.13)	<.001	13.12 (12.91, 13.34)	8.71 (8.49, 8.93)	−4.41 (−4.72, −4.11)	<.001	−5.34 (−5.71, −4.98)	<.001
Median	6	6	–	–	12	7	–	–	–	–
Variance	25.96	34.43	–	–	53.64	43.61	–	–	–	–
% Abnormal	5.60% (4.92, 6.37)	8.89% (8.29, 9.51)	3.29% (2.44, 3.99)	<.001	31.77% (30.41, 33.15)	14.23% (13.11, 15.43)	−17.54% (−19.33, −15.75)	<.001	–	–
**Emotional problems**										
Mean	1.48 (1.43, 1.53)	1.85 (1.80, 1.89)	0.37 (0.30, 0.43)	<.001	4.19 (4.11, 4.27)	2.69 (2.61, 2.77)	−1.50 (−1.62, −1.39)	<.001	−1.87 (−1.99, −1.74)	<.001
Median	1	1	–	–	4	2	–	–	–	–
Variance	3.04	3.98	–	–	7.37	5.47	–	–	–	–
% Abnormal	7.26% (6.53, 8.06)	10.95% (10.3, 11.6)	3.69% (2.85, 4.55)	<.001	41.75% (40.30, 43.21)	20.11% (18.82, 21.47)	−21.64% (−23.61, −19.67)	<.001	–	–
**Conduct problems**										
Mean	1.26 (1.22, 1.31)	1.38 (1.34, 1.41)	0.12 (0.06, 0.17)	<.001	2.50 (2.43, 2.57)	1.40 (1.34, 1.46)	−1.10 (−1.19, −1.01)	<.001	−1.22 (−1.32, −1.11)	<.001
Median	1	1	–	–	2	1	–	–	–	–
Variance	2.18	2.52	–	–	5.38	3.40	–	–	–	–
% Abnormal	8.22% (7.41, 9.12)	9.81% (9.19, 10.47)	1.59% (0.70, 2.44)	<.001	31.15% (29.80, 32.53)	13.18% (12.10, 14.35)	−17.97% (−19.73, 16.20)	<.001	–	–
**Hyperactive problems**										
Mean	2.86 (2.80, 2.93)	3.11 (3.06, 3.16)	0.25 (0.16, 0.33)	<.001	4.32 (4.23, 4.41)	3.22 (3.13, 3.32)	1.10 (−1.23, −0.96)	<.001	−1.35 (−1.50, 1.18)	<.001
Median	3	3	–	–	4	2	–		–	–
Variance	5.09	6.09	–	–	9.59	8.78	–		–	–
% Abnormal	7.59% (6.83, 8.43)	10.39% (9.75, 11.06)	2.80% (1.94, 3.64)	<.001	26.30% (24.98, 27.58)	16.30% (15.12, 17.56)	−10.00% (−11.74, −8.18)	<.001	–	–
**Peer problems**										
Mean	1.16 (1.11, 1.20)	1.34 (1.31, 1.38)	0.18 (0.13, 0.25)	<.001	2.11 (2.05, 2.16)	1.39 (1.33, 1.45)	−0.72 (−0.80, −0.63)	<.001	−0.90 (−1.00, −0.80)	<.001
Median	1	1	–	–	2	1	–		–	–
Variance	2.49	2.89	–	–	3.85	3.04	–		–	–
% Abnormal	9.05% (8.22, 9.95)	11.13% (10.48, 11.81)	2.08% (1.17, 2.99)	<.001	25.20% (23.95, 26.50)	13.55% (12.46, 14.72)	−11.65% (−13.36, 9.94)	<.001	–	–

a Changes refer to mean differences between continuous scores that were compared over time within each country using *t*-tests, and across country and time using an interaction between country and time. The percentage of abnormal scores were binary outcomes that were compared within each country using proportions tests. Estimates from ALSPAC use entropy balanced weights that were added as an interaction with the inverse probability weights. For the MCS, sample design weights were added as an interaction with the inverse probability weights. For both Pelotas cohorts, inverse probability weights were used.

**Figure 2.**
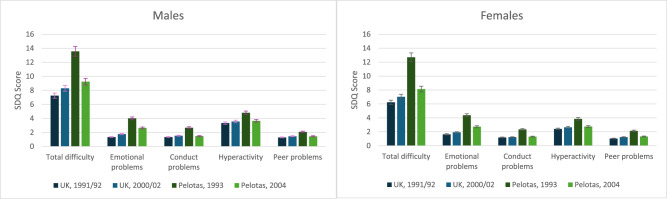
Differences in SDQ total and subscale scores at age 11 among males and females in the UK and Pelotas cohorts. Note total difficulty scores range from 0 to 40, and individual subscales from 0 to 10.

There were some differences over time period in mental health problems among females and males in the UK (Fig. 2). Increases across the two cohorts in conduct problems were greater for males relative to females, and for emotional problems, males experienced greater increases over time compared to females at age 11 (see Supplementary Table 11).

In Brazil, total difficulty scores decreased for adolescents born in the 2000s compared to those born in the 1990s (see Fig. 1). Specifically, mean total difficulty scores decreased by −4.41 (−4.72, −4.11 for 11-year-olds, representing a medium effect (Cohens *d* = 0.40). Decreases over the time period were also found for individual subscales, with declines greatest for emotional problems at 11 years (see Table 1). The percentage of adolescents scoring in the abnormal range for emotional problems dropped from 41.7% in 1993 to 20.1% in 2004.

Comparisons between males and females in Pelotas showed decreasing emotional and peer problems over time were larger for females compared to males, while decreases in conduct problems were greater for males (see Fig. 2 and Supplementary Table 11).

#### Cross-country comparisons of mental health at age 11

Cross-country comparisons confirmed differences between the UK and Brazil for total difficulties over the same time period, and each individual subscale of mental health problems, with all two way interactions of country and cohort significant. The largest cross-national differences in trends were for differences in emotional problems at age 11 (UK increasing, Brazil decreasing). When comparing sex differences across countries over the same time period, analyses revealed that males aged 11 experienced greater increases over time in emotional and conduct problems compared to females in the UK, whereas in Pelotas, males experienced greater decreases over time (See Supplementary Table 11). The opposite was found for peer problems at age 11, whereby females experienced greater increases over time compared to males in the UK, while in Pelotas, females experienced greater decreases compared to males.

### Follow-up analyses

Similar cross-country differences were found when comparing scores in later adolescence. In the UK, increases over the time period were found for total difficulty, emotional, conduct and peer problem scores over time when comparing both 14 and 17 year olds (See Supplementary Tables 13 and 14). Increases were greater than those noted at 11 years for total difficulty and emotional problems. One observed difference between the two adolescent age groups was that 14-year-olds born in the early 2000s had *higher* parent-reported hyperactive problems compared to those reported for 14-year-olds born in the early 1990s (Supplementary Table 14): the opposite to that found for 17-years-olds (Supplementary Table 13). In addition, unlike the findings at age 11, females in adolescence experienced greater increases in emotional problems over time compared to males (Supplementary Table 15). In Pelotas, findings were highly consistent and showed reduced scores in the more recent cohort for the total difficulty scale and all four subscales.

## Discussion

This study represents the first to compare two population-based cohorts in the UK, and two population-based cohorts in Pelotas, Brazil, to understand how mental health problems differ over the same time period across the two settings. In doing so, our study provides the most up-to-date insight into population-level rates of youth mental health problems in Pelotas, Brazil, and unique insight into how these have changed over the last two decades in comparison to the UK.

### Differences in adolescent mental health problems

In the UK, parent-rated SDQ total difficulty scores, as well as scores on the individual subscales, were higher for adolescents who were born more recently. These findings align with similar studies conducted in high-income countries showing increases in adolescent mental health problems, and in particular emotional problems (Armitage *et al.*, 2023a), and converge with surveys using diagnostic measures of psychopathology (Sadler *et al.*, 2018).

Findings from the Pelotas cohorts in Brazil suggest that increasing rates of youth mental health problems over this period are not universal. Total difficulty and subscale scores were lower among young people in Pelotas born more recently. Similar declines over time periods have been noted for conduct problems among 4-year-olds in the Pelotas cohorts (Degli Esposti *et al.*, 2023).

Despite promising overall reductions in mental health problems in Pelotas, SDQ scores remained higher in the most recent Pelotas cohort compared to the UK: around 20.11% of adolescents in Pelotas scored in the abnormal range for emotional problems at 11 years, compared to 10.95% in the UK. This finding aligns with other studies that have compared internalising problems across the UK and Brazil (Moltrecht *et al.*, 2024).

### Explaining cross-country differences in adolescent mental health problems

There are various possible explanations for the cross-country differences over the time periods studied. One is that the reduction in problems in Pelotas reflects broader economic, social and epidemiological changes that have occurred in recent decades (Bertodi *et al.*, 2019; Degli Esposti *et al.*, 2023). During this period, Brazil experienced many positive social and environmental changes, including increased schooling and reductions in absolute income-related inequalities (Degli Esposti *et al.*, 2023). Previous research in Brazil found that improvements in socioeconomic conditions may have counteracted otherwise increasing suicide rates (Machaldo *et al.*, 2015), with regions experiencing larger decreases in income inequality also having greater declines in adolescent suicide. Regions exposed to decreased rates of suicide were those in the South and Center-West regions of Brazil, mirroring the declines in mental health in the South observed in the present study. These combined findings highlight the importance of social policies aimed at improving the living conditions of young people to prevent mental health problems.

The rise in mental health problems in the UK and other high-income countries has been attributed to a number of factors, including increased inequalities (Anthony *et al.*, 2023; Collishaw *et al.*, 2019), changes to lifestyle, academic stress, digital media, weight and weight-related concerns (Collishaw and Sellers, 2020; Gage *et al.*, 2021; Högberg *et al.*, 2020; Twenge *et al.*, 2018). Research in Brazil has revealed some secular trends following this pattern – with sleep quality (Hoefelmann *et al.*, 2013), physical fitness (Nevill *et al.*, 2023) and physical activity (Mielke *et al.*, 2014) of children and adolescents declining since the early 2000s, and body dissatisfaction increasing (Gonzaga *et al.*, 2023); however, little is known about changes in school-related stress and the use of digital media over time. Further research is necessary to understand population-level drivers of trends across the two countries.

An important consideration when comparing rates of mental health between countries relates to measurement differences. Although measurement invariance was established across time within each country, informant rating thresholds and interpretation of questions are inherently subjective and influenced by cultural norms. Future research using more objective approaches to measurement of mental health will be critical in addressing these issues (Bluett-Duncan *et al.*, 2024).

It is also important to recognise that social and cultural influences may have changed across time. For example, there may be increased help-seeking by parents and young people due to improved screening and clinical recognition in schools and primary care, as well as changes to perceptions of what constitutes a mental health difficulty (Collishaw, 2015). There is some evidence that learning about psychiatric concepts may increase mental health problems, which has led some to argue that mental health awareness efforts may be contributing to the rise in mental health problems (Foulkes and Andrews, 2023). Understanding how attitudes have changed over time will be a challenge; however, it remains an important priority to understanding cross-country differences in mental health over time.

### Limitations

Findings should be interpreted in light of some limitations. First, to ensure comparability across the four cohorts, parent-reports of mental health were used. Parents may under-report their child’s emotional problems, particularly during adolescence, as some symptoms may not always be aware to others. This means that our estimates may be conservative; however, this is less likely to influence estimated changes over time. Second, results in the ALSPAC cohort were weighted to be representative of the national population at that time. This enabled comparison with the other national cohort in the UK, the MCS. This was not necessary in Pelotas as both were from the same population. However, this means results may not be generalisable to children living elsewhere in Brazil, who may experience variations in economic inequality, health and crime rates (Brito *et al.*, 2022; Cerqueira *et al.*, 2021). In addition, there were some differences between the two Pelotas cohorts in how the SDQ was administered. In 1993, this was carried out by lay persons trained by a psychologist, while in 2004, assessments were conducted by trained psychologists. Such approaches differ to the UK assessments, which were completed as questionnaires. Finally, selective attrition occurred over time periods and country. Inverse probability weighting was used to make samples more representative of the baseline population but this does not account for unmeasured factors that systematically influence missingness.

### Implications

Our findings suggest that increasing secular trends in youth mental health problems documented in several developed countries may not be representative across all countries. Further research is now necessary to understand why secular trends may vary across countries. Such research should consider a combination of quantitative and qualitative methods to shed light on these differences, and ensure that approaches are sensitive to the context in which they are based.

### Conclusions

This study represents the first population-based comparison of adolescent mental health problems across different countries over the same time period. We show that while mental health problems increased over time in the UK, and declined over a similar time period in Pelotas, they remained higher in Pelotas compared to in the UK. Our findings shed light on a growing need to address changing mental health problems and gaps between the UK and Brazil. Overall, the difference in cross-cohort change represents a valuable opportunity to examine putative contributors to trends in youth mental health.

## Supporting information

Armitage et al. supplementary material Armitage et al. supplementary material

## Data Availability

The data that support the findings of this study are available on request. The data are not publicly available due to privacy or ethical restrictions.

## Supplementary material

The supplementary material for this article can be found at https://doi.org/10.1017/S2045796025000137.

## References

[ref1] Anselmi L, Fleitlich-Bilyk B, Menezes AMB, Arau´jo CL and Rohde LA (2010) Prevalence of psychiatric disorders in a Brazilian birth cohort of 11-year-olds. *Social Psychiatry & Psychiatric Epidemiology* 45, 135–142. 19381426 10.1007/s00127-009-0052-2

[ref2] Anthony R, Moore G, Page N, Ollerhead C, Parker J, Murphy S, Rice F, Armitage JM and Collishaw S (2023) Trends in adolescent emotional problems in Wales between 2013 and 2019: The contribution of peer relationships. *Journal of Child Psychology and Psychiatry*. doi:10.1111/jcpp.13924. 38083987

[ref3] Armitage JM, Kwong ASF, Tseliou F, Sellers R, Blakey R, Anthony R, Rice F, Thapar A and Collishaw S (2023a) Cross-cohort change in parent-reported emotional problem trajectories across childhood and adolescence in the UK. *The Lancet Psychiatry* 10(7), 509–517. 37244272 10.1016/S2215-0366(23)00175-X

[ref4] Armitage JM, Tseliou F, Riglin L, Dennison C, Eyre O, Bevan-Jones R, Rice F, Thapar AK, Thapar A and Collishaw S (2023b) Validation of the Strengths and Difficulties Questionnaire (SDQ) emotional subscale in assessing depression and anxiety across development. *PLoS ONE* 18(7), e0288882. 10.1371/journal.pone.0288882 PMC10355443 37467238

[ref5] Baranne ML and Falissard B (2018) Global burden of mental disorders among children aged 5-14 years. *Child and Adolescent Psychiatry and Mental Health* 12, 19. 10.1186/s13034-018-0225-4 PMC5896103 29682005

[ref6] Bertoldi AD, Barros FC, Hallal PRC, Mielke GI, Oliveira PD, Maia MFS, Horta BL, Gonçalves H, Barros AJD, Tovo-Rodrigues L, Murray J and Victora CG (2019) Trends and inequalities in maternal and child health in a Brazilian city: Methodology and sociodemographic description of four population-based birth cohort studies, 1982–2015. *International Journal of Epidemiology* 48(Supplement_1), i4–i15. 30883654 10.1093/ije/dyy170 PMC6422064

[ref7] Bluett-Duncan M, Pickles A, Chandra PS, Hill J, Kishore MT, Satyanarayana V and Sharp H (2024) Experience and Reporting of Postnatal Depression Across Cultures: A Comparison Using Anchoring Vignettes of Mothers in the United Kingdom and India. *American Journal of Epidemiology* 193(1), 214–226. 37667811 10.1093/aje/kwad182 PMC10773478

[ref8] Boyd A, Golding J, Macleod J, Lawlor DA, Fraser A, Henderson J, Molloy L, Ness A, Ring S and Davey Smith G (2013) Cohort Profile: The ‘children of the 90s’–the index offspring of the Avon Longitudinal Study of Parents and Children. *International Journal of Epidemiology* 42(1), 111–127. 22507743 10.1093/ije/dys064 PMC3600618

[ref9] Brito VCDA, Bello-Corassa R, Stopa SR, Sardinha LMV, Dahl CM and Viana MC (2022) Prevalence of self-reported depression in Brazil: National health survey 2019 and 2013. *Epidemiologia e Serviços de Saúde, Brasília* 31(1), e2021384. 10.1590/SS2237-9622202200006.especial PMC9897827 35830090

[ref10] Cerqueira D, Ferreira H, Bueno S, Alves P, Lima RSD, Marques D, Silva FABD, Lunelli IC, Rodrigues RI, Lins GDOA, Armstrong KC, Lira P, Coelho D, Barros B, Sobral I, Pacheco D and Pimentel A (2021) Atlas da Violência FBSP. São Paulo:IPEA – Instituto de Pesquisa Econômica Aplicada.

[ref11] Collishaw S (2015) Annual research review: Secular trends in child and adolescent mental health. *Journal of Child Psychology and Psychiatry* 56(3), 370–393. 25496340 10.1111/jcpp.12372

[ref12] Collishaw S, Furzer E, Thapar AK and Sellers R (2019) Brief report: A comparison of child mental health inequalities in three UK population cohorts. *European Child and Adolescent Psychiatry* 28(11), 1547–1549. 30848392 10.1007/s00787-019-01305-9 PMC6800845

[ref13] Collishaw S, and Sellers R (2020) Trends in Child and Adolescent Mental Health Prevalence, Outcomes, and Inequalities. In Taylor E., Verhulst F., Wong J., Yoshida K. and Nikapota A. (edited by), *Mental Health and Illness of Children and Adolescents. Mental Health and Illness Worldwide*. Singapore: Springer, 63–73.

[ref14] Connelly R and Platt L (2014) Cohort profile: UK Millennium Cohort Study (MCS). *International Journal of Epidemiology* 43(6), 1719–1725. 24550246 10.1093/ije/dyu001

[ref15] Degli Esposti M, Matijasevich A, Collishaw S, Martins-Silva T, Santos IS, Baptista Menezes AM, Domingues MR, Wehrmeister FC, Barros F and Murray J (2023) Secular trends and social inequalities in child behavioural problems across three Brazilian cohort studies (1993, 2004 and 2015). *Epidemiology and Psychiatric Sciences* 32, e23. 10.1017/S2045796023000185 PMC10130841 37066785

[ref16] Foulkes L and Andrews JL (2023) Are mental health awareness efforts contributing to the rise in reported mental health problems? A call to test the prevalence inflation hypothesis. *New Ideas in Psychology* 69, 101010.

[ref17] Fraser A, Macdonald-Wallis C, Tilling K, Boyd A, Golding J, Davey Smith G, Henderson J, Macleod J, Molloy L, Ness A, Ring S, Nelson SM and Lawlor DA (2013) Cohort Profile: The Avon Longitudinal Study of Parents and Children: ALSPAC mothers cohort. *International Journal of Epidemiology* 42(1), 97–110. 22507742 10.1093/ije/dys066 PMC3600619

[ref18] Gage SH and Patalay P (2021) Associations between adolescent mental health and health-related behaviors in 2005 and 2015: A population cross-cohort study. *The Journal of Adolescent Health: Official Publication of the Society for Adolescent Medicine* 69(4), 588–596. 33867232 10.1016/j.jadohealth.2021.03.002

[ref19] Gonçalves H, Assunção MC, Wehrmeister FC, Oliveira IO, Barros FC, Victora CG, Hallal PC and Menezes AM (2014) Cohort profile update: The 1993 Pelotas (Brazil) birth cohort follow-up visits in adolescence. *International Journal of Epidemiology* 43(4), 1082–1088. 24729426 10.1093/ije/dyu077 PMC4121560

[ref20] Gonzaga I, Ribovski M, Claumann GS, Folle A, Beltrame TS, Laus MF and Pelegrini A (2023) Secular trends in body image dissatisfaction and associated factors among adolescents (2007–2017/2018). *PloS One* 18(1), e0280520. 10.1371/journal.pone.0280520 PMC9851498 36656894

[ref21] Hoefelmann LP, da Silva Lopes A, da Silva KS, Moritz P and Nahas MV (2013) Sociodemographic factors associated with sleep quality and sleep duration in adolescents from Santa Catarina, Brazil: What changed between 2001 and 2011? *Sleep Medicine* 14(10), 1017–1023. 23890599 10.1016/j.sleep.2013.05.015

[ref22] Högberg B, Strandh M and Hagquist C (2020) Gender and secular trends in adolescent mental health over 24 years - The role of school-related stress. *Social Science & Medicine* 250(1982), 112890. 10.1016/j.socscimed.2020.112890 32143086

[ref23] Machado DB, Rasella D and Dos Santos DN (2015) Impact of income inequality and other social determinants on suicide rate in Brazil. *PloS One* 10(4), e0124934. 10.1371/journal.pone.0124934 PMC4416030 25928359

[ref24] Mari J (2014) Mental healthcare in Brazil: Modest advances and major challenges. *Advances in Psychiatric Treatment* 20(2), 113–115.

[ref25] Matijasevich A, Murray E, Stein A, Anselmi L, Menezes AM, Santos IS, Barros AJD, Gigante DP, Barros FC and Victora CG (2014) Increase in child behavior problems among urban Brazilian 4‐year olds: 1993 and 2004 Pelotas birth cohorts. *Journal of Child Psychology and Psychiatry* 55(10), 1125–1134. 24735354 10.1111/jcpp.12236 PMC4263231

[ref26] Mielke GI, Hallal PC, Malta DC and Lee IM (2014) Time trends of physical activity and television viewing time in Brazil: 2006-2012. *The International Journal of Behavioral Nutrition and Physical Activity* 11, 101. 10.1186/s12966-014-0101-4 PMC4149267 25124462

[ref27] Moltrecht B, Villanova Do Amaral J, Salum GA, Miguel EC, Rohde LA, Ploubidis GB, McElroy E and Hoffmann MS (2024) Social connection and its prospective association with adolescent internalising and externalising symptoms: An exploratory cross-country study using retrospective harmonisation. *Journal of Child Psychology and Psychiatry*. Advance online publication. doi: 10.1111/jcpp.14080. PMC12018293 39644141

[ref28] Nevill AM, Duncan MJ, Gaya A and Mello JB (2023) Secular trends in the physical fitness of Brazilian youth: Evidence that fitness is declining for the majority but not for a fit minority. *Scandinavian Journal of Medicine and Science in Sports* 33(10), 2079–2089. 37403435 10.1111/sms.14440

[ref29] Plewis L (2007). The Millennium Cohort Study: Technical report on sampling. Centre for Longitudinal Studies, London.

[ref30] Sadler K, Vizard T, Ford T, Goodman A, Goodman R and McManus S (2018) *Mental Health of Children and Young People in England, 2017: Trends and Characteristics*. Leeds, UK: NHS Digital.

[ref31] Santos IS, Barros AJ, Matijasevich A, Domingues MR, Barros FC and Victora CG (2011) Cohort profile: The 2004 Pelotas (Brazil) birth cohort study. *International Journal of Epidemiology* 40(6), 1461–1468. 20702597 10.1093/ije/dyq130 PMC3235016

[ref32] Thapar A, Eyre O, Patel V and Brent D (2022) Depression in young people. *The Lancet* 400(10352), 617–631. 10.1016/S0140-6736(22)01012-1 35940184

[ref33] Twenge JM, Martin GN and Campbell WK (2018) Decreases in psychological well-being among American adolescents after 2012 and links to screen time during the rise of smartphone technology. *Emotion* 18, 765–780. 29355336 10.1037/emo0000403

[ref34] Victora CG, Hallal PC, Araújo CLP, Menezes AMB, Wells JCK and Barros FC (2008) Cohort Profile: The 1993 Pelotas (Brazil) Birth Cohort Study. *International Journal of Epidemiology* 37(4), 704–709. 17846051 10.1093/ije/dym177

