# Supplementary material for: A cross-country comparison of temporal change in adolescent mental health problems in the UK and Brazil
Source: Epidemiol Psychiatr Sci. 2025 Mar 12;34:e17. doi: 10.1017/S2045796025000137 (PMC11955427; doi:10.1017/S2045796025000137)
Supplement: Armitage et al. supplementary material [file S2045796025000137sup001.docx]

**Supplementary material:**

**A cross country comparison of temporal change in adolescent mental health problems in the UK and Brazil**

Armitage, J. M., Viegas da Silva, E., Tseliou, F., Riglin, L., Hammerton, G., Collishaw, S., Santos, I., Tovo-Rodrigues, L., Menezes, A., Wehrmeister, F., Gonçalves, H., Matijasevich, A., & Murray, J.

**Supplementary Methods**

*Sample weights to enhance representativeness and comparability of the cohorts*

In ALSPAC, weights were generated aiming to represent the UK population, using data from the 1991 Census Household Sample of Anonymised Records for Great Britain (SARs). This is a 1% representative sample of the total population in Great Britain and Northern Ireland at that time. We used information from mothers with an infant less than 1 years old to ensure comparability with the ALSPAC cohort (see Supplementary Table 5). An entropy balancing weight value was generated for all ALSPAC participants based on variables related to ethnicity, home ownership, and maternal education. Entropy balancing is a multivariate, reweighting method designed to balance two samples on prespecified variables.

*Measurement invariance - methods*

Measurement invariance was assessed using structural equation modelling, and was carried out on each age point separately across the two countries. Five factors representing each of the SDQ subscales were included in one model, each indexed by the relevant 5 ordinal items. We modelled increasingly stringent types of measurement invariance, modelling item loadings and item thresholds sequentially (Bowen & Masa, 2015) using the automated measurement invariance testing in Mplus (MODEL = configural metric scalar) (Muthén & Muthén, 1998-2017). The configural invariance model specified the same pattern of loadings across earlier and later cohorts (each of the twenty-five items loading onto one of the five factors, with loadings freely estimated across cohort). The metric (“weak”) invariance model fixed item loadings to be the same across cohort, and the scalar (“strong”) invariance model fixed thresholds to be the same across cohort. Freed and fixed parameters for each model are outlined in Supplementary Table 6 (Muthén & Muthén, 1998-2017). Models were fit using delta parameterisation with a robust weighted least squares estimator (WLSMV) including individuals with partially incomplete data (within or between cohorts). Model fit was assessed using the comparative fit index (CFI), the root-mean-square error of approximation (RMSEA) and the standardized root mean squared residual (SRMSR), with values of ≥0.95, ≤0.06 and ≤0.08 (respectively) generally considered indicative of good fit (Hu & Bentler, 1999). Change in CFI was the criteria used to accept models. All measurement invariance analyses were conducted in Mplus version 8 (Muthén & Muthén, 1998-2017).

*Measurement invariance - results*

For both countries, at both ages (11 and 15-17 years), we found evidence of strong scalar invariance such that model fit did not notably decrease (ΔCFI<-0.01) when fixing factor loadings across earlier and later cohorts (metric compared to configural models), or when subsequently fixing threshold loadings to be equal across cohorts (scalar compared to metric models) (see Supplementary Table 7). All four scalar models (UK age 11, UK age 17, Pelotas age 11, Pelotas age 15) showed good model fit according to the RMSEA and SRMR, although CFI values fell below the recommended value of ≥0.95. Factor loadings for age 11 and age 15 in the UK and Pelotas cohorts are shown in Supplementary Table 8. Based on these findings, analyses continued using the total difficulty scores and individual subscales as sum scales (rather than latent variables).

**References**

- Bowen, N. K., & Masa, R. D. (2015). Conducting measurement invariance tests with ordinal data: A guide for social work researchers*. Journal of the Society for Social Work and Research, 6*(2), 229–249.
- Hu, L. T., & Bentler, P. M. (1999). Cutoff Criteria for Fit Indexes in Covariance Structure Analysis: Conventional Criteria Versus New Alternatives. Structural Equation Modeling-a Multidisciplinary Journal, 6(1), 1–55. <https://doi.org/10.1080/10705519909540118>
- K., & Muthén, B. O. (n.d.). *Mplus User’s Guide* (Eighth). Muthén & Muthén.
- Putnick, D. L., & Bornstein, M. H. (2016). Measurement invariance conventions and reporting: The state of the art and future directions for psychological research. *Developmental Review, 41,* 71–90

**Supplementary Tables**

| **Supplementary Table 1: SDQ response rates across cohorts** | | | | |
| --- | --- | --- | --- | --- |
|  | **UK** | | **Brazil** | |
|  | **ALSPAC** | **MCS** | **1993 Pelotas cohort** | **2004 Pelotas cohort** |
| N at baseline | 15,442 | 18,577 | 5,249 | 4,231 |
| Age at assessment | 11 years | 11 years | 11 years | 11 years |
| Year at assessment | 2002/03 | 2012/13 | 2004 | 2015 |
| N with complete SDQ (%) | 7,257 (47%) | 12,420 (67%) | 4,423 (84%) | 3,563 (84%) |
|  | **ALSPAC** | **MCS** | **1993 Pelotas cohort** | **2004 Pelotas cohort** |
| Age at assessment | 17 years | 17 years | 15 years | 15 years |
| Year at assessment | 2008/09 | 2018 | 2008 | 2019 |
| N with complete SDQ (%) | 5,553 (36%) | 7,748 (42%) | 4,338 (83%) | 1,942 (46%) |
| *Sensitivity analyses*  *(UK only)* |  |  |  |  |
| Age at assessment | 14 years | 14 years | - | - |
| Year at assessment | 2004/2006 | 2015/16 | - | - |
| N with complete SDQ (%) | 6,939 (45%) | 11,404 (61%) | - | - |

| **Supplementary Table 2: Variables used for inverse probability weighting** | | | | | | | | |
| --- | --- | --- | --- | --- | --- | --- | --- | --- |
|  | **UK** | | | | **Brazil** | | | |
|  | **ALSPAC** | | **MCS** | | **1993 Pelotas cohort** | | **2004 Pelotas cohort** | |
|  | **Variable** | **Age assessed** | **Variable** | **Age assessed** | **Variable** | **Age assessed** | **Variable** | **Age assessed** |
| Birthweight | Continuous  (grams) | Perinatal | Continuous  (grams) | 9 months  (subsample) | Continuous (grams) | Perinatal | Continuous (grams) | Perinatal |
| Premature | Yes/No | Perinatal | Any complications? Yes premature | 9 months | Yes/No | Perinatal | Yes/No | Perinatal |
| Multiple pregnancies | Single/Multiple | Perinatal | One or two | 9 months | One or two | Perinatal |  |  |
| Mother age | Continuous | 18 weeks gestation | Continuous | 9 months | Continuous | Perinatal | Continuous | Perinatal |
| Mother has partner/ lives with partner | 0=Never Married/ Widowed/ Divorced  1=Married (first or second) | 8 months | 1=Legally separated; 2=Married 1^st^ and only; 3=Remarried 2^nd^ or later; 4=Single never married; 5=Divorced; 6=Widowed | 9 months | Lives with partner:  0=Yes; 1=No | Perinatal | Lives with partner:  0=Yes; 1=No | Perinatal |
| Home ownership status | 0=Council rented/Private rented/Private rented/HA rented/Other  1=Buying/mortgage/own outright | 8 months | 0=Rent from local authority/Rent from HA/Rent privately/Living with parents/Live rent free/Squatting/Other  1=Own outright/Mortgage/loan/3=Part rent, part mortgage | 9 months | - | - | - | - |
| Mother ethnic group/skin colour | 0=Black Caribbean/Black African/Indian/Pakistani/Bangladeshi/Chinese/Other  1=White | 32 weeks gestation | 0= Mixed/Indian/Pakistani and Bangladeshi/ Black or Black British/Other/Chinese and other,  1=White | 9 months | 0=Black or other  1=White | Perinatal | 0=Black or other  1=White | Perinatal |
| Mother worked full or part-time during pregnancy | 0=Yes; 1=No | 32 weeks gestation | 0=Yes; 1=No | 9 months | 0=Yes; 1=No | Perinatal | 0=Yes; 1=No | Perinatal |
| Mother academic schooling | 0=No degree, 1=Degree | 32 weeks gestation | 0=No degree, 1=Degree | 9 months | Continuous | Perinatal | Continuous | Perinatal |
| Mother smoking during pregnancy | 0=No; 1=Yes | 18 weeks gestation | 0=No; 1=Yes | 9 months | 0=No, 1=Yes | Perinatal | 0=No, 1=Yes | Perinatal |
| Mother mental health | EPDS | 8, 21, and 33 months | Kessler Scale | 3 years | Self-reporting questionnaire (SRQ) | 11 years | EPDS | 3, 12, 24, and 48 months |
| Household income | Continuous based on each week, including social benefits | 11 years | Total net income bands -annual based on couple | 9 months and 11 years | Continuous based on day? | Perinatal | Continuous based on month (use quintile) | Perinatal |
| Note: Where data were not assessed in some cohorts, these sections have been left blank. | | | | | | | | |

| **Supplementary Table 3: Comparison of baseline characteristics across UK cohorts** | | | | | |
| --- | --- | --- | --- | --- | --- |
|  | **Weighted** | | **Unweighted** | | **Difference between unweighted** |
|  | **ALSPAC**  (%) | **MCS**  (%) | **ALSPAC**  (%) | **MCS**  (%) | (*x^2^ , p-*value) |
| Premature (<37 weeks) |  |  |  |  | 869.01, <0.001 |
| No | 92.2% | 99.7% | 92.6% | 99.7% |  |
| Yes | 7.8% | 0.3% | 7.4% | 0.3% |  |
| Multiple pregnancies |  |  |  |  | 2.54, 0.11 |
| No | 98.9% | 98.6% | 98.7% | 98.5% |  |
| Yes | 1.1% | 1.4% | 1.3% | 1.5% |  |
| Child’s sex |  |  |  |  | 0.25, 0.62 |
| Male | 51.6% | 51.4% | 51.1% | 51.4% |  |
| Female | 48.4% | 48.6% | 48.9% | 48.6% |  |
| Mother skin colour |  |  |  |  | 1.40, <0.001 |
| White | 95.3% | 87.6% | 97.4% | 84.2% |  |
| Black/other | 4.7% | 12.4% | 2.6% | 15.8% |  |
| Maternal age |  |  |  |  | 48.31, <0.001 |
| ≤19 | 4.9% | 5.8% | 4.2% | 5.9% |  |
| 20-34 | 86.2% | 74.5% | 76.7% | 74.7% |  |
| ≥35 | 8.9% | 19.7% | 19.1% | 19.4% |  |
| Mother years of schooling |  |  |  |  | 40.97, <0.001 |
| No degree | 94.1% | 84.6% | 87.1% | 84.5% |  |
| Degree | 5.9% | 15.4% | 12.9% | 15.5% |  |
| Mother married |  |  |  |  | 1.30, <0.001 |
| Yes | 70.0% | 58.5% | 79.5% | 59.2% |  |
| No | 30.0% | 41.5% | 20.5% | 40.8% |  |
| Housing tenure |  |  |  |  | 749.9, <0.001 |
| Mortgage/own house | 58.1% | 59.0% | 73.2% | 58.3% |  |
| Renting/other | 41.9% | 41.0% | 26.8% | 41.7% |  |
| Mother worked during pregnancy |  |  |  |  | 1.50, <0.001 |
| Yes | 29.7% | 62.2% | 34.6% | 62.3% |  |
| No | 71.3% | 37.8% | 65.4% | 37.7% |  |
| Household income (quintiles) |  |  |  |  | 86.1, <0.001 |
| Poorest | 26.7% | 19.1% | 20.0% | 22.7% |  |
| 2^nd^ | 24.1% | 19.1% | 22.7% | 19.9% |  |
| 3^rd^ | 21.3% | 23.8% | 22.4% | 23.1% |  |
| 4^th^ | 17.3% | 18.4% | 20.6% | 17.1% |  |
| Richest | 10.6% | 19.6% | 14.3% | 17.2% |  |
| Mother smoked during pregnancy |  |  |  |  | 110.91, <0.001 |
| No | 70.8% | 69.0% | 74.8% | 69.4% |  |
| Yes | 29.2% | 31.0% | 25.2% | 30.6% |  |
| Mother mental health problems |  |  |  |  | 1.37, 0.24 |
| No | 70.7% | 70.7% | 71.9% | 71.2% |  |
| Yes | 29.3% | 29.3% | 28.1% | 28.8% |  |
| Note: Mother mental health problems were assessed in ALSPAC using the Edinburgh Postnatal Depression Scale (EPDS), while in the MCS mothers were asked if they had either been diagnosed with depression or anxiety. Both assessments took place when the child was 3 years old. Mental problems in the EPDS are defined as scores of 10 or above. | | | | | |

| **Supplementary Table 4: Comparison of baseline characteristics across Brazil cohorts** | | | |
| --- | --- | --- | --- |
|  | **Pelotas 1993**  (%) | **Pelotas 2004**  (%) | **Difference**  ( *x^2^ , p-*value ) |
| Low birthweight (<2500g) |  |  | 0.01, 0.91 |
| No | 89.8% | 89.8% |  |
| Yes | 10.2% | 10.2% |  |
| Premature (<37 weeks) |  |  | 18.29, <0.001 |
| No | 88.5% | 85.5% |  |
| Yes | 11.5% | 14.5% |  |
| Multiple pregnancies |  |  | 75.47, <0.001 |
| No | 94.5% | 98.0% |  |
| Yes | 5.5% | 2.0% |  |
| Child’s sex |  |  | 4.87, 0.05 |
| Male | 49.6% | 51.9% |  |
| Female | 50.4% | 48.1% |  |
| Mother skin colour |  |  | 23.56, <0.001 |
| White | 77.3% | 73.0% |  |
| Black/other | 22.7% | 27.0% |  |
| Maternal age |  |  | 18.76, <0.001 |
| ≤19 | 17.4% | 18.9% |  |
| 20-34 | 71.6% | 67.7% |  |
| ≥35 | 11.0% | 13.4% |  |
| Mother years of schooling |  |  | 375.26, <0.001 |
| 0-4 | 28.0% | 15.6% |  |
| 5-8 | 46.2% | 41.4% |  |
| ≥9 | 25.8% | 43.0% |  |
| Mother lives with partner |  |  | 31.18, <0.001 |
| Yes | 87.6% | 83.6% |  |
| No | 12.4% | 16.4% |  |
| Mother worked during pregnancy |  |  | 8.75, <0.001 |
| Yes | 37.1% | 40.0% |  |
| No | 62.9% | 60.0% |  |
| Household income (quintiles) |  |  | 16.17, <0.01 |
| Poorest | 20.1% | 20.6% |  |
| 2^nd^ | 23.2% | 20.2% |  |
| 3^rd^ | 17.3% | 19.3% |  |
| 4^th^ | 19.5% | 20.3% |  |
| Richest | 19.9% | 19.6% |  |
| Mother smoked during pregnancy |  |  | 38.30< 0.001 |
| No | 66.6% | 72.5% |  |
| Yes | 33.4% | 27.5% |  |
| Mother mental health problems |  |  | 3.44, 0.06 |
| No | 69.1% | 71.0% |  |
| Yes | 30.9% | 29.0% |  |
| Note: Mother mental health problems were assessed using the Self-report questionnaire (SRQ) in the Pelotas 1993 cohort when children were 11 years, and using the Edinburgh Postnatal Depression Scale (EPDS) in the Pelotas 2004 cohort when children were 2 years. Mental problems in the SRQ are defined as scores of 8 or above, and in EPDS as scores of 10 or above. | | | |

| **Supplementary Table 5: Comparison of characteristics between ALSPAC (1991-92), 1991 census, and MCS (2000-02)** | | | | | |
| --- | --- | --- | --- | --- | --- |
|  | **ALSPAC**  **(raw)** | **ALSPAC**  **(entropy balanced)** | **1991 - UK**  **(n=28,089)** | **1991 – England**  **(n=24,338)** | **MCS - UK**  **(raw)** |
| Ethnicity of mother |  |  |  |  |  |
| White | 97.4% | 90.5% | 90.5% | 89.4% | 84.2% |
| Non-White | 2.6% | 9.5% | 9.5% | 10.6% | 15.8% |
| Housing tenure |  |  |  |  |  |
| Own outright/Own buying | 73.2% | 63.0% | 63.0% | 64.4% | 58.3% |
| Renting/other | 26.8% | 37.0% | 37.0% | 35.6% | 41.7% |
| Mother education |  |  |  |  |  |
| Degree | 87.1% | 92.4% | 92.4% | 92.5% | 84.5% |
| No degree | 12.9% | 7.6% | 7.6% | 7.5% | 15.5% |
| Note: Data from 1991 Census based on women with an infant <1 year of age. The household sample of anonymised record (SAR) was used over the individual SAR so that the age of youngest dependent child in household variable could be used. Entropy balancing was done using the UK cohort. | | | | | |

| **Supplementary Table 6**: **Freed and fixed parameters for measurement invariance models** | | | | | |
| --- | --- | --- | --- | --- | --- |
| **Model** | **Factor loadings** | **Thresholds** | **Scale factors*** | **Factors means** | **Factor variances** |
| Configural | Free across groups | Free across groups | Fixed at one in both groups | Fixed at zero in both groups | Free across groups |
| Metric | Equal across groups | First item thresholds and first threshold for subsequent items equal across groups; second thresholds free | Fixed at one in the first group; free in the second group | Fixed at zero in the first group; free in the second group | Free across groups |
| Scalar | Equal across groups | Equal across groups | Fixed at one in the first group; free in the second group | Fixed at zero in the first group; free in the second group | Free across groups |
| Note: *Scale factors are functions of the factor loadings, the factor variances, and the residual variances.  Groups refer to the cohorts for whom measurement invariance is being tested, i.e. the Avon Longitudinal Study of Parents and Children (ALSPAC), the Millenium Cohort Study (MCS), Pelotas 1993 and Pelotas 2004. | | | | | |

| **Supplementary Table 7. Tests of measurement invariance within-country across time: Full SDQ model** | | | | | | | | | | |
| --- | --- | --- | --- | --- | --- | --- | --- | --- | --- | --- |
| Model | Free parameters | CFI | RMSEA (90% CI) | SRMR | vs. | Δ parameters | ΔCFI | ΔRMSEA | ΔSRMR | Decision |
| ***UK: Age 11*** | | | | | | | | | | |
| M1a: Configural invariance | 170 | 0.915 | 0.053 (0.053-0.055) | 0.062 | - | - | - | - | - | - |
| M2a: Metric invariance | 150 | 0.912 | 0.054 (0.054-0.055) | 0.063 | M1a | -20 | -0.003 | +0.001 | +0.001 | Accept |
| M3a: Scalar invariance | 130 | 0.911 | 0.054 (0.053-0.054) | 0.063 | M2a | -20 | -0.001 | 0.000 | 0.000 | Accept |
| ***UK: Age 17*** | | | | | | | | | | |
| M1c: Configural invariance | 170 | 0.907 | 0.058 (0.058-0.059) | 0.070 | - | - | - | - | - | - |
| M2b: Metric invariance | 150 | 0.904 | 0.058 (0.058-0.059) | 0.071 | M1b | -20 | -0.03 | 0.000 | +0.001 | Accept |
| M3b: Scalar invariance | 130 | 0.905 | 0.057 (0.056-0.057) | 0.072 | M2b | -20 | +0.001 | -0.001 | +0.001 | Accept |
| ***Pelotas: Age 11*** | | | | | | | | | | |
| M1c: Configural invariance | 170 | 0.898 | 0.053 (0.052-0.054) | 0.071 | - | - | - | - | - | - |
| M2c: Metric invariance | 150 | 0.899 | 0.051 (0.050-0.053) | 0.071 | M1c | -20 | +0.001 | -0.002 | 0.000 | Accept |
| M3c: Scalar invariance | 130 | 0.893 | 0.052 (0.053-0.053) | 0.072 | M2c | -20 | -0.005 | +0.001 | +0.001 | Accept |
| ***Pelotas: Age 15*** | | | | | | | | | | |
| M1d: Configural invariance | 170 | 0.875 | 0.059 (0.058-0.060) | 0.078 | - | - | - | - | - | - |
| M2d: Metric invariance | 150 | 0.872 | 0.059 (0.057-0.060) | 0.079 | M1d | -20 | -0.003 | 0.000 | +0.001 | Accept |
| M3d: Scalar invariance | 130 | 0.872 | 0.057 (0.056-0.059) | 0.080 | M1d | -20 | 0.000 | -0.002 | +0.001 | Accept |
| Accepted where ΔCFI<-0.01. | | | | | | | | | | |

| **Supplementary Table 8: Unstandardised factor loadings: scalar invariance models** | | | | |
| --- | --- | --- | --- | --- |
|  | UK, Age 11 | UK, Age 17 | Pelotas, Age 11 | Pelotas, Age 15 |
| Emotional problems |  |  |  |  |
| Often complains of headaches… | 1 | 1 | 1 | 1 |
| Many worries… | 1.831 | 1.812 | 0.548 | 0.619 |
| Often unhappy, downhearted… | 1.932 | 1.866 | 1.598 | 1.718 |
| Nervous or clingy in new situations… | 1.584 | 1.667 | 1.188 | 1.219 |
| Many fears, easily scared | 1.781 | 1.830 | 1.216 | 1.229 |
| Conduct problems |  |  |  |  |
| Often has temper tantrums or hot tempers | 1 | 1 | 1 | 1 |
| Generally obedient… | -1.026 | -0.943 | -0.937 | -0.945 |
| Often fights with other children… | 1.109 | 1.011 | 0.975 | 0.886 |
| Often lies or cheats | 1.049 | 1.098 | 0.955 | 0.962 |
| Steals from home, school or elsewhere | 1.038 | 1.068 | 0.741 | 0.779 |
| Hyperactivity |  |  |  |  |
| Restless, overactive… | 1 | 1 | 1 | 1 |
| Constantly fidgeting or squirming | 0.958 | 1.139 | 0.970 | 0.979 |
| Easily distracted, concentration wanders | 1.018 | 1.120 | 0.927 | 0.983 |
| Thinks things out before acting | -0.928 | -1.118 | -0.775 | -0.914 |
| Sees tasks through to the end | -1.012 | -1.160 | -0.845 | -0.954 |
| Peer problems |  |  |  |  |
| Rather solitary, tends to play alone | 1 | 1 | 1 | 1 |
| Has at least one good friend | -1.051 | -1.123 | -1.526 | -1.508 |
| Generally liked by other children | -1.377 | -1.482 | -2.246 | -1.959 |
| Picked on or bullied by other children… | 1.267 | 1.236 | 1.978 | 1.679 |
| Gets on better with adults than with other children | 1.042 | 0.973 | 1.423 | 1.168 |
| Prosocial behaviour |  |  |  |  |
| Considerate of other people's feelings | 1 | 1 | 1 | 1 |
| Shares readily with other children… | 0.845 | 0.850 | 0.842 | 0.728 |
| Helpful if someone is hurt… | 0.814 | 0.868 | 0.744 | 0.737 |
| Kind to younger children | 0.839 | 0.781 | 0.827 | 0.758 |
| Often volunteers to help others… | 0.645 | 0.711 | 0.584 | 0.574 |

| **Supplementary Table 9. Tests of measurement invariance across countries: Full SDQ model** | | | | | | | | | | |
| --- | --- | --- | --- | --- | --- | --- | --- | --- | --- | --- |
| Model | Free parameters | CFI | RMSEA (90% CI) | SRMR | vs. | Δ parameters | ΔCFI | ΔRMSEA | ΔSRMR | Decision |
| ***Age 11*** | | | | | | | | | | |
| M1a: Configural invariance | 170 | 0.908 | 0.056 (0.056–0.057) | 0.065 | - | - | - | - | - | - |
| M2a: Metric invariance | 150 | 0.868 | 0.066 (0.066-0.067) | 0.071 | M1a | -20 | -0.04 | 0.010 | 0.006 | Accept |
| M3a: Scalar invariance | 130 | 0.850 | 0.069 (0.069–0.070) | 0.078 | M2a | -20 | -0.018 | 0.003 | 0.007 | Accept |
| ***Age 15/17*** | | | | | | | | | | |
| M1c: Configural invariance | 170 | 0.895 | 0.061 (0.060-0.062) | 0.072 | - | - | - | - | - | - |
| M2c: Metric invariance | 150 | 0.876 | 0.065 (0.064-0.066) | 0.077 | M1c | -20 | -0.019 | 0.004 | 0.005 | Accept |
| M3c: Scalar invariance | 130 | 0.869 | 0.066 (0.065-0.066) | 0.079 | M2c | -20 | -0.007 | 0.001 | 0.002 | Accept |
| Accepted where ΔCFI<-0.01. | | | | | | | | | | |

| **Supplementary Table 10: Unstandardised factor loadings: scalar invariance from across countries models** | | |
| --- | --- | --- |
|  | Age 11 | Age 15/17 |
| Emotional problems |  |  |
| Often complains of headaches… | 1.00 | 1.00 |
| Many worries… | 1.695 | 1.611 |
| Often unhappy, downhearted… | 1.523 | 1.480 |
| Nervous or clingy in new situations… | 1.443 | 1.476 |
| Many fears, easily scared | 1.656 | 1.569 |
| Conduct problems |  |  |
| Often has temper tantrums or hot tempers | 1.00 | 1.00 |
| Generally obedient… | 0.989 | 0.773 |
| Often fights with other children… | 1.074 | 0.980 |
| Often lies or cheats | 1.038 | 1.056 |
| Steals from home, school or elsewhere | 0.942 | 0.906 |
| Hyperactivity |  |  |
| Restless, overactive… | 1.00 | 1.00 |
| Constantly fidgeting or squirming | 1.028 | 1.102 |
| Easily distracted, concentration wanders | 1.060 | 1.129 |
| Thinks things out before acting | 0.795 | 0.824 |
| Sees tasks through to the end | 1.005 | 1.047 |
| Peer problems |  |  |
| Rather solitary, tends to play alone | 1.00 | 1.00 |
| Has at least one good friend | 1.116 | 1.152 |
| Generally liked by other children | 1.341 | 1.258 |
| Picked on or bullied by other children… | 1.135 | 1.096 |
| Gets on better with adults than with other children | 1.018 | 0.927 |
| Prosocial behaviour |  |  |
| Considerate of other people's feelings | 1.00 | 1.00 |
| Shares readily with other children… | 0.934 | 0.863 |
| Helpful if someone is hurt… | 1.104 | 1.060 |
| Kind to younger children | 1.014 | 0.910 |
| Often volunteers to help others… | 0.903 | 0.858 |

| **Supplementary Table 11: Cross country comparison of mean and abnormal SDQ scores at age 11 years (unweighted)** | | | | | | | |
| --- | --- | --- | --- | --- | --- | --- | --- |
|  | **UK** | | | **Brazil** | | |  |
|  | **ALSPAC**  **1991-92** | **MCS**  **2000-02** | **Change in UK** ^Ϯ^ | **Pelotas cohort**  **1993** | **Pelotas cohort**  **2004** | **Change in Brazil** ^Ϯ^ | **Difference between changes over time in UK and changes over time in Brazil** |
| 11 years | Mean  (95% CI) | Mean  (95% CI) | Estimate | Mean  (95% CI) | Mean  (95% CI) | Estimate | Difference |
| **Total difficulty score** | | | | | | | |
| Mean | 6.56  (6.45, 6.68) | 7.73  (7.63, 7.83) | 1.17 ***  (1.01, 1.33) | 13.14  (12.93, 13.36) | 8.64  (8.43, 8.86) | -4.50 ***  (-4.81, -4.19) | -5.67 ***  (-5.98, -5.35) |
| Median | 5 | 6 | - | 12 | 7 | - |  |
| Variance | 24.82 | 34.20 | - | 53.72 | 43.27 | - |  |
| % Abnormal | 4.9% | 8.9% | 4.0% *** | 31.9% | 14.0% | -17.9% *** |  |
| **Emotional problem score** | | | | | | | |
| Mean | 1.47  (1.43, 1.51) | 1.87  (1.83, 1.90) | 0.40 ***  (0.34, 0.45) | 4.20  (4.12, 4.28) | 2.68  (2.60, 2.75) | -1.52 ***  (-1.64, -1.41) | -1.92 ***  (-2.03, -1.81) |
| Median | 1 | 1 | - | 4 | 2 | - |  |
| Variance | 3.01 | 3.97 | - | 7.37 | 5.45 | - |  |
| % Abnormal | 7.1% | 11.1% | 4.0% *** | 41.9% | 19.9% | -22.5% *** |  |
| **Conduct problem score** | | | | | | | |
| Mean | 1.21  (1.18, 1.24) | 1.38  (1.36, 1.41) | 0.17 ***  (0.13, 0.22) | 2.51  (2.43, 2.58) | 1.39  (1.33, 1.45) | -1.12 ***  (-1.21, -1.02) | -1.29 ***  (-1.38, -1.20) |
| Median | 1 | 1 | - | 2 | 1 | - |  |
| Variance | 2.04 | 2.47 | - | 5.40 | 3.37 | - |  |
| % Abnormal | 7.4% | 9.8% | 2.4% *** | 31.2% | 13.0% | -18.2% *** |  |
| **Hyperactive score** | | | | | | | |
| Mean | 2.78  (2.73, 2.84) | 3.12  (3.08, 3.17) | 0.34 ***  (0.27, 0.41) | 4.32  (4.23, 4.41) | 3.21  (3.11, 3.30) | -1.11 ***  (-1.25, -0.98) | -1.45 ***  (-1.59, -1.32) |
| Median | 2 | 3 | - | 4 | 2 | - |  |
| Variance | 5.01 | 6.15 | - | 9.58 | 8.74 | - |  |
| % Abnormal | 7.2% | 10.5% | 3.3% *** | 26.3% | 16.1% | -10.2% *** |  |
| **Peer problems score** | | | | | | | |
| Mean | 1.12  (1.08, 1.16) | 1.37  (1.34, 1.40) | 0.25 ***  (0.21, 0.30) | 2.11  (2.06, 2.17) | 1.37  (1.32, 1.43) | -0.74 ***  (-0.82, -0.66) | -0.99 ***  (-1.08, -0.90) |
| Median | 1 | 1 | - | 2 | 1 | - |  |
| Variance | 2.44 | 2.86 | - | 3.86 | 3.01 | - |  |
| % Abnormal | 8.8% | 11.5% | 2.7% *** | 25.3% | 13.3% | -12.0% *** |  |
| ***p<.001, **p<0.01, *p<.05  Note: ^Ϯ^ Changes refer to mean differences between continuous scores that were compared over time within each country using t-tests, and across country and time using an interaction between country and time. The percentage of abnormal scores were binary outcomes that were compared within each country using proportions tests. | | | | | | | |

| **Supplementary Table 12: Comparison of mean and abnormal SDQ scores at age 11 years by sex** | | | | | | | | | | |
| --- | --- | --- | --- | --- | --- | --- | --- | --- | --- | --- |
|  | **UK** | | | | **Brazil** | | | |  | |
|  | **ALSPAC**  **1991-92** | **MCS**  **2000-02** | **Change in UK** | | **Pelotas cohort**  **1993** | **Pelotas cohort**  **2004** | **Change in Brazil** |  | **Difference between changes over time in UK and changes over time in Brazil** | |
| **11 years** |  |  |  |  |  |  |  |  |  |  |
| **Total SDQ** | Mean  (95% CI) | Mean  (95% CI) | Estimate | P value | Mean  (95% CI) | Mean  (95% CI) | Estimate | P value | Difference | P value |
| **Males** | | | | | | | | | | |
| Mean | 7.25  (7.02, 7.48) | 8.29  (8.10, 8.48) | 1.04  (0.74, 1.34) | <0.001 | 13.56  (13.26, 13.87) | 9.24  (8.93, 9.55) | -4.32  (-4.76, -3.88) | <0.001 | -5.36  (-5.89, -4.83) | <0.001 |
| Median | 6 | 7 | - | - | 13 | 8 | - | - | - | - |
| Variance | 29.01 | 38.11 | - | - | 53.75 | 46.39 | - | - | - | - |
| % Abnormal | 7.14%  (6.11, 8.38) | 10.84%  (9.94, 11.81) | 3.70%  (2.50, 4.90) | <0.001 | 34.59%  (32.62, 36.62) | 16.59%  (14.94, 18.38) | -18.0%  (-20.63, -15.38) | <0.001 | - | - |
| **Females** | | | | | | | | | | |
| Mean | 6.23  (6.04, 6.43) | 7.03  (6.87, 7.20) | 0.80  (0.54, 1.06) | <0.001 | 12.69  (12.39, 12.99) | 8.14  (7.84, 8.44) | -4.55  (-4.98, -4.13) | <0.001 | 5.35  (-5.85, -4.85) | <0.001 |
| Median | 5 | 6 | - | - | 12 | 7 | - | - | - | - |
| Variance | 22.49 | 29.94 | - | - | 53.19 | 40.03 | - | - | - | - |
| % Abnormal | 4.10%  (3.33, 5.07) | 6.91%  (6.17, 7.74) | 2.81%  (1.87, 3.77) | <0.001 | 29.01%  (27.17, 30.93) | 11.72%  (10.27, 13.35) | -17.29%  (-19.71, -14.87) | <0.001 | - | - |
| **Time x sex** | - | - | -0.24  (-0.63, 0.15) | 0.230 | - | - | -0.23  (-0.84, 0.38) | 0.46 | - | - |
| **Country x sex x time** | - | - | - | - | - | - | - | - | 0.01  (-0.72, 0.74) | 0.97 |
| **Emotional problems** | | | | | | | | | | |
| **Males** | | | | | | | | | | |
| Mean | 1.34  (1.27, 1.41) | 1.77  (1.71, 1.83) | 0.43  (0.34, 0.53) | <0.001 | 4.01  (3.89, 4.12) | 2.64  (2.53, 2.75) | -1.37  (-1.52, -1.21) | <0.001 | -1.80  (-1.98, -1.62) | <0.001 |
| Median | 1 | 1 | - | - | 4 | 2 | - | - | - | - |
| Variance | 2.85 | 3.93 | - | - | 7.24 | 5.51 | - | - | - | - |
| % Abnormal | 6.17%  (5.23, 7.27) | 10.47%  (9.59, 11.41) | 4.30%  (3.15, 5.45) | <0.001 | 39.07%  (37.04, 41.14) | 19.15%  (17.40, 21.03) | -19.92%  (-22.64, -17.18) | <0.001 | - | - |
| **Females** | | | | | | | | | | |
| Mean | 1.63  (1.55, 1.70) | 1.92  (1.86, 1.98) | 0.30  (0.20, 0.38) | <0.001 | 4.37  (4.26, 4.49) | 2.74  (2.63, 2.85) | -1.63  (-1.79, -1.47) | <0.001 | -1.93  (-2.11, -1.74) | <0.001 |
| Median | 1 | 1 | - | - | 4 | 2 | - | - | - | - |
| Variance | 3.18 | 4.02 | - | - | 7.43 | 5.43 | - | - | - | - |
| % Abnormal | 8.32%  (7.24, 9.55) | 11.43%  (10.50, 12.45) | 3.11%  (1.85, 4.27) | <0.001 | 44.36%  (42.32, 46.43) | 21.14%  (19.26, 23.15) | -23.22%  (-26.05, -20.39) | <0.001 | - | - |
| **Time x sex** | - | - | -0.14  (-0.27, -0.01) | <0.05 | - | - | -0.27  (-0.49, -0.05) | <0.05 | - | - |
| **Country x sex x time** | - | - | - | - | - | - | - | - | -0.13  (-0.39, 0.13) | 0.34 |
| **Conduct problems** | | | | | | | | | | |
| **Males** | | | | | | | | | | |
| Mean | 1.34  (1.27, 1.41) | 1.53  (1.47, 1.58) | 0.19  (0.10, 0.27) | <0.001 | 2.67  (2.57, 2.77) | 1.48  (1.40, 1.57) | -1.19  (-1.32, -1.05) | <0.001 | -1.38  (-1.53, -1.22) | <0.001 |
| Median | 1 | 1 | - |  | 2 | 1 | - | - | - | - |
| Variance | 2.51 | 2.90 | - |  | 5.60 | 3.58 | - | - | - | - |
| % Abnormal | 9.83%  (8.61, 11.21) | 11.91%  (10.97, 12.92) | 2.08%  (0.75, 3.4% | <0.01 | 33.78%  (31.82, 35.79) | 14.33%  (12.79, 16.03) | - 19.45%  (-22.00, -16.89) | <0.001 | - | - |
| **Females** | | | | | | | | | |  |
| Mean | 1.19  (1.13, 1.25) | 1.23  (1.18, 1.27) | 0.04  (-0.04, 0.11) | <0.001 | 2.34  (2.24, 2.43) | 1.31  (1.23, 1.40) | -1.03  (-1.15, -0.90) | <0.001 | -1.07  (-1.21, -0.91) | <0.001 |
| Median | 1 | 1 | - | - | 2 | 1 | - | - | - | - |
| Variance | 1.84 | 2.09 | - | - | 5.12 | 3.20 | - | - | - | - |
| % Abnormal | 6.65%  (5.63, 7.83) | 7.70%  (6.93, 8.55) | 1.05%  (0.58, 2.15) | 0.07 | 28.59%  (26.76, 30.50) | 11.96%  (10.49, 13.60) | -16.63%  (-19.05, -14.21) | <0.001 | - | - |
| **Time x sex** | - | - | -0.15  (-0.26, -0.04) | <0.01 | - | - | 0.17  (-0.02, 0.35) | 0.08 | - | - |
| **Country x sex x time** | - | - | - | - | - | - | - |  | 0.32  (0.10, 0.53) | <0.01 |
| **Hyperactive problems** | | | | | | | | | | |
| **Males** | | | | | | | | | | |
| Mean | 3.33  (3.22, 3.43) | 3.56  (3.56, 3.64) | 0.23  (0.11, 0.36) | <0.001 | 4.81  (4.68, 4.94) | 3.66  (3.52, 3.80) | -1.15  (-1.34, -0.95) | <0.001 | -1.38  (-1.61, -1.15) | <0.001 |
| Median | 3 | 3 | - | - | 5 | 3 | - | - | - | - |
| Variance | 5.66 | 6.69 | - | - | 9.60 | 9.54 | - | - | - | - |
| % Abnormal | 10.46%  (9.23, 11.84) | 14.12%  (13.10, 15.20) | 3.66%  (2.27, 5.04) | <0.001 | 31.31%  (29.40, 33.29) | 21.09%  (19.27, 23.03) | -10.22%  (-12.92, -7.52) | <0.001 | - | - |
| **Females** | | | | | | | | | | |
| Mean | 2.41  (2.32, 2.50) | 2.65  (2.58, 2.72) | 0.24  (0.13, 0.35) | <0.001 | 3.85  (3.72, 3.97) | 2.76  (2.63, 2.89) | -1.09  (-1.27, -0.91) | <0.001 | -1.33  (-1.54, -1.12) | <0.001 |
| Median | 2 | 2 | - | - | 4 | 2 | - | - | - | - |
| Variance | 4.12 | 5.08 | - | - | 9.12 | 7.55 | - | - | - | - |
| % Abnormal | 4.78%  (3.94, 5.80) | 6.66%  (5.92, 7.44) | 1.86%  (0.88, 2.84) | <0.001 | 21.34%  (19.70, 23.09) | 11.19%  (9.78, 12.78) | -10.15%  (-12.42, -7.89) | <0.001 | - | - |
| **Time x sex** | - | - | 0.00  (-0.16, 0.17) | 0.97 | - | - | 0.05  (-0.21, 0.32) | 0.69 | - | - |
| **Country x sex x time** | - | - | - | - | - | - | - |  | 0.05  (-0.26, 0.36) | 0.75 |
| **Peer problems** | | | | | | | | | | |
| **Males** | | | | | | | | | | |
| Mean | 1.29  (1.21, 1.36) | 1.45  (1.39, 1.50) | 0.16  (0.07, 0.25) | <0.001 | 2.08  (2.00, 2.16) | 1.45  (1.37, 1.53) | -0.63  (-0.74, -0.51) | <0.001 | -0.79  (-0.93, -0.64) | <0.001 |
| Median | 1 | 1 | - | - | 2 | 1 | - | - | - | - |
| Variance | 2.84 | 3.20 | - | - | 3.66 | 3.17 | - | - | - | - |
| % Abnormal | 10.60%  (9.34, 12.01) | 12.62%  (11.67, 13.64) | 2.02%  (0.66, 3.39) | <0.01 | 24.44%  (22.68, 26.29) | 15.55  (13.93, 17.28) | -8.89%  (-11.36, -6.46) | <0.001 | - | - |
| **Females** | | | | | | | | | | |
| Mean | 1.03  (0.97, 1.09) | 1.24  (1.19, 1.29) | 0.21  (0.13, 0.29) | <0.001 | 2.13  (2.05, 2.22) | 1.33  (1.24, 1.41) | -0.80  (-0.92, -0.69) | <0.001 | -1.01  (-1.16, -0.88) | <0.001 |
| Median | 0 | 1 | - | - | 2 | 1 | - | - | - | - |
| Variance | 2.12 | 2.57 | - | - | 4.04 | 2.89 | - | - | - | - |
| % Abnormal | 7.53%  (6.50, 8.70) | 9.63%  (8.77, 10.56) | 2.10%  (0.91, 3.29) | <0.001 | 25.95%  (24.18, 27.80) | 11.45%  (10.01, 13.06) | -14.50%  (-16.87, -12.14) | <0.001 | - | - |
| **Time x sex** | - | - | 0.05  (-0.07, 0.17) | 0.39 | - | - | -0.18  (-0.34, -0.02) | <0.05 | - | - |
| **Country x sex x time** | - | - | - | - | - | - | - | - | -0.23  (-0.43, -0.03) | <0.05 |
| Note: ^Ϯ^ Changes refer to mean differences between continuous scores that were compared over time within each country using t-tests, and across country and time using an interaction between country and time. The percentage of abnormal scores were binary outcomes that were compared within each country using proportions tests. Estimates from ALSPAC use entropy balanced weights that were added as an interaction with the inverse probability weights. For the MCS, sample design weights were added as an interaction with the inverse probability weights. For both Pelotas cohorts, inverse probability weights were used. | | | | | | | | | | |

| **Supplementary Table 13: Comparison of mean and abnormal SDQ scores at 15/17 year** | | | | | | | | | | |
| --- | --- | --- | --- | --- | --- | --- | --- | --- | --- | --- |
|  | **UK** | | | | **Brazil** | | | |  | |
|  | **ALSPAC**  **1991-92** | **MCS**  **2000-02** | **Change in UK**^Ϯ^ | | **Pelotas cohort**  **1993** | **Pelotas cohort**  **2004** | **Change in Brazil**^Ϯ^ | | **Difference between changes over time in UK and changes over time in Brazil** | |
| **15/17 years** | Mean  (95% CI) | Mean  (95% CI) | Estimate | P value | Mean  (95% CI) | Mean  (95% CI) | Estimate | P value | Difference† | P value |
| **Total SDQ** | | | | | | | | |  |  |
| Mean | 6.40  (6.22, 6.57) | 7.50  (7.33, 7.66) | 1.10  (0.86, 1.34) | <.001 | 12.10  (11.89, 12.32) | 8.99  (8.69, 9.29) | -3.11  (-3.48, 2.75) | <.001 | -4.21  (-4.65, -3.78) | <.001 |
| Median | 5 | 6 | - | - | 11 | 8 | - | - | - | - |
| Variance | 23.53 | 36.24 | - | - | 50.82 | 43.92 | - | - | - | - |
| % Abnormal | 4.77%  (4.04,5.62) | 9.22%  (8.45, 10.06) | 4.45%  (4.29, 4.60) | <.001 | 26.90%  (25.60, 28.24) | 14.98%  (13.44, 16.67) | 11.92%  (9.85, 13.99) | <.001 | - | - |
| **Emotional problems** | | | | | | | | |  |  |
| Mean | 1.54  (1.48, 1.61) | 2.08  (2.02, 2.15) | 0.54  (0.45, 0.63) | <.001 | 3.94  (3.86, 4.02) | 3.21  (3.09, 3.32) | -0.73  (-0.87, -0.59) | <.001 | -1.27  (-1.44, -1.11) | <.001 |
| Median | 1 | 1 | - | - | 4 | 3 | - | - | - | - |
| Variance | 3.46 | 5.27 | - | - | 7.22 | 6.62 | - | - | - | - |
| % Abnormal | 8.39%  (7.46, 9.42) | 15.56%  (14.59, 16.59) | 7.17%  (6.05, 8.31) | <.001 | 37.96%  (36.53, 39.42) | 27.93%  (25.95, 29.99) | -10.03%  (-12.51, -7.57) | <.001 | - | - |
| **Conduct problems** | | | | | | | | |  |  |
| Mean | 1.09  (1.04, 1.14) | 1.15  (1.11, 1.19) | 0.06  (-0.00, 0.13) | 0.06 | 2.28  (2.22, 2.35) | 1.43  (1.34, 1.51) | -0.85  (-0.96, -0.75) | <.001 | -0.91  (-1.15, -0.79) | <.001 |
| Median | 1 | 1 | - | - | 2 | 1 | - | - | - | - |
| Variance | 2.02 | 2.21 | - | - | 5.12 | 3.27 | - | - | - | - |
| % Abnormal | 6.32%  (5.48, 7.29) | 7.35%  (6.65, 8.10) | 1.03%  (0.12, 1.92) | 0.03 | 26.77%  (25.47, 28.11) | 13.15%  (11.68, 14.76) | -13.62%  (-15.63, -11.63) | <.001 | - | - |
| **Hyperactive problems** | | | | | | | | |  |  |
| Mean | 2.67  (2.60, 2.75) | 2.48  (2.42, 2.54) | -0.19  (-0.29, -0.10) | <.001 | 3.80  (3.71, 3.89) | 2.63  (2.51, 2.75) | -1.17  (-1.32, -1.02) | <.001 | -0.96  (-1.15, -0.79) | <.001 |
| Median | 2 | 2 | - | - | 4 | 2 | - | - | - | - |
| Variance | 4.65 | 5.23 | - | - | 9.34 | 7.19 | - | - | - | - |
| % Abnormal | 6.23%  (5.41, 7.17) | 6.34%  (5.70, 7.04) | 0.11  (-0.00, 0.10) | 0.805 | 20.72%  (19.54, 21.96) | 10.93%  (9.59, 12.43) | -9.79%  (-11.63, -7.95) | <.001 | - | - |
| **Peer problems** | | | | | | | | |  |  |
| Mean | 1.15  (1.10, 1.20) | 1.79  (1.73, 1.84) | 0.64  (0.56, 0.71) | <.001 | 2.09  (2.03, 2.14) | 1.72  (1.64, 1.81) | -0.37  (-0.46, -0.26) | <.001 | -1.01  (-1.12, -0.87) | <.001 |
| Median | 1 | 1 | - | - | 2 | 1 | - | - | - | - |
| Variance | 2.21 | 3.45 | - | - | 3.85 | 3.37 | - | - | - | - |
| % Abnormal | 7.40%  (6.56, 8.34) | 16.53%  (15.53, 17.57) | 9.13%  (8.00, 10.24) | <.001 | 25.78%  (24.50, 27.11) | 17.08  (15.45, 18.84) | -8.70%  (-10.83, -6.58) | <.001 | - | - |
| Note: ^Ϯ^ Changes refer to mean differences between continuous scores that were compared over time within each country using t-tests, and across country and time using an interaction between country and time. The percentage of abnormal scores were binary outcomes that were compared within each country using proportions tests. Estimates from ALSPAC use entropy balanced weights that were added as an interaction with the inverse probability weights. For the MCS, sample design weights were added as an interaction with the inverse probability weights. For both Pelotas cohorts, inverse probability weights were used. | | | | | | | | | | |

| **Supplementary Table 14: Comparison of mean and abnormal SDQ scores using age 14 data in UK cohorts** | | | | | | | | | | | |
| --- | --- | --- | --- | --- | --- | --- | --- | --- | --- | --- | --- |
|  | **UK** | | | | **Brazil** | | | |  | | |
|  | **ALSPAC**  **1991-92** | **MCS**  **2000-02** | **Change in UK** ^Ϯ^ | | **Pelotas cohort**  **1993** | **Pelotas cohort**  **2004** | **Change in Brazil** ^Ϯ^ | | **Difference between changes over time in UK and changes over time in Brazil** | | |
| **14 years** | Mean  (95% CI) | Mean  (95% CI) | Estimate | P value | Mean  (95% CI) | Mean  (95% CI) | Estimate | P value | Difference† | Robust SE | P value |
| **Total SDQ** | | | | | | | | |  |  | |
| Mean | 7.04  (6.88, 7.21) | 8.52  (8.39, 8.65) | 1.48  (1.27, 1.68) | <.001 | 12.10  (11.89, 12.32) | 8.99  (8.69, 9.29) | -3.11  (-3.48, 2.75) | <.001 | -4.59  (-4.17, -5.01) | 0.21 | <.001 |
| Median | 6 | 7 | - | - | 11 | 8 | - | - | - | - | - |
| Variance | 27.12 | 30.24 | - | - | 50.82 | 43.92 | - | - | - | - | - |
| % Abnormal | 6.20%  (5.55, 7.04) | 9.46%  (8.80, 10.17) | 3.26%  (2.43, 4.09) | <.001 | 26.90%  (25.60, 28.24) | 14.98%  (13.44, 16.67) | 11.92%  (9.85, 13.99) | <.001 | - | - | - |
| **Emotional problems** | | | | | | | | |  |  | |
| Mean | 1.46  (1.41, 1.52) | 2.06  (2.01, 2.11) | 0.60  (0.52, 0.67) | <.001 | 3.94  (3.86, 4.02) | 3.21  (3.09, 3.32) | -0.73  (-0.87, -0.59) | <.001 | -1.31  (-1.48, -1.17) | 0.08 | <.001 |
| Median | 1 | 1 | - | - | 4 | 3 | - | - | - | - | - |
| Variance | 3.08 | 4.62 | - | - | 7.22 | 6.62 | - | - | - | - | - |
| % Abnormal | 6.91%  (6.18, 7.73) | 13.54%  (12.76, 14.50) | 6.63%  (5.70, 7.55) | <.001 | 37.96%  (36.53, 39.42) | 27.93%  (25.95, 29.99) | -10.03%  (-12.51, -7.57) | <.001 | - | - | - |
| **Conduct problems** | | | | | | | | |  |  | |
| Mean | 1.33  (1.28, 1.38) | 1.41  (1.37, 1.44) | 0.08  (0.01, 0.14) | < .05 | 2.28  (2.22, 2.35) | 1.43  (1.34, 1.51) | -0.85  (-0.96, -0.75) | <.001 | -0.93  (-1.06, -0.81) | 0.06 | <.001 |
| Median | 1 | 1 | - | - | 2 | 1 | - | - | - | - | - |
| Variance | 2.29 | 2.73 | - | - | 5.12 | 3.27 | - | - | - | - | - |
| % Abnormal | 8.70%  (7.84, 9.64) | 10.39%  (9.70, 11.13) | 1.69%  (0.77, 2.62) | <.001 | 26.77%  (25.47, 28.11) | 13.15%  (11.68, 14.76) | -13.62%  (-15.63, -11.63) | <.001 | - | - | - |
| **Hyperactive problems** | | | | | | | | |  |  | |
| Mean | 3.02  (2.95, 3.08) | 3.34  (3.30, 3.38) | 0.32  (0.24, 0.40) | <.001 | 3.80  (3.71, 3.89) | 2.63  (2.51, 2.75) | -1.17  (-1.32, -1.02) | <.001 | -1.49  (-1.66, -1.32) | 0.09 | <.001 |
| Median | 3 | 3 | - | - | 4 | 2 | - | - | - | - | - |
| Variance | 5.07 | 3.41 | - | - | 9.34 | 7.19 | - | - | - | - | - |
| % Abnormal | 8.19%  (7.38, 8.09) | 7.30%  (6.72, 7.93) | -0.89%  (-0.17, -0.00) | <.05 | 20.72%  (19.54, 21.96) | 10.93%  (9.59, 12.43) | -9.79%  (-11.63, -7.95) | <.001 | - | - | - |
| **Peer problems** | | | | | | | | |  |  | |
| Mean | 1.26  (1.21, 1.31) | 1.72  (1.68, 1.77) | 0.46  (0.40, 0.53) | <.001 | 2.09  (2.03, 2.14) | 1.72  (1.64, 1.81) | -0.37  (-0.46, -0.26) | <.001 | -0.83  (-0.95, -0.71) | 0.06 | <.001 |
| Median | 1 | 1 | - | - | 2 | 1 | - | - | - | - | - |
| Variance | 2.71 | 3.37 | - | - | 3.85 | 3.37 | - | - | - | - | - |
| % Abnormal | 10.49%  (9.58, 11.48) | 15.72%  (14.89, 16.58) | 5.23%  (4.18, 6.27) | <.001 | 25.78%  (24.50, 27.11) | 17.08  (15.45, 18.84) | -8.70%  (-10.83, -6.58) | <.001 | - | - | - |
| Note: ^Ϯ^ Changes refer to mean differences between continuous scores that were compared over time within each country using t-tests, and across country and time using an interaction between country and time. The percentage of abnormal scores were binary outcomes that were compared within each country using proportions tests. Estimates from ALSPAC use entropy balanced weights that were added as an interaction with the inverse probability weights. For the MCS, sample design weights were added as an interaction with the inverse probability weights. For both Pelotas cohorts, inverse probability weights were used. | | | | | | | | | | | |

| **Supplementary Table 15 Comparison of mean and abnormal SDQ scores at age 15/17 years by sex** | | | | | | | | | | |
| --- | --- | --- | --- | --- | --- | --- | --- | --- | --- | --- |
|  | **UK** | | | | **Brazil** | | | |  | |
|  | **ALSPAC**  **1991-92** | **MCS**  **2000-02** | **Change in UK** ^Ϯ^ | | **Pelotas cohort**  **1993** | **Pelotas cohort**  **2004** | **Change in Brazil** ^Ϯ^ | | **Difference between changes over time in UK and changes over time in Brazil** | |
| **15/17 years** |  |  |  |  |  |  |  |  |  |  |
| **Total SDQ** | Mean  (95% CI) | Mean  (95% CI) | Estimate | P value | Mean  (95% CI) | Mean  (95% CI) | Estimate | P value | Difference | P value |
| **Males** | | | | | | | | | | |
| Mean | 6.29  (6.05, 6.53) | 7.54  (7.30, 7.78) | 1.25  (0.91, 1.60) | <0.001 | 11.86  (11.56, 12.16) | 8.67  (8.26, 9.08) | -3.19  (-3.71, -2.67) | <0.001 | -4.44  (-5.06, -3.83) | <0.001 |
| Median | 5 | 6 | - | - | 11 | 7 | - | - |  |  |
| Variance | 22.68 | 37.46 | - | - | 49.71 | 42.78 | - | - |  |  |
| % Abnormal | 4.90%  (3.84, 6.24) | 9.72%  (8.61, 10.9) | 4.82%  (3.52, 6.12) | <0.001 | 25.30%  (23.49, 27.19) | 14.20%  (12.13, 16.57) | -11.10%  (-13.94, 8.24) | <0.001 |  | <0.001 |
| **Females** | | | | | | | | | | |
| Mean | 6.49  (6.25, 6.73) | 7.45  (7.22, 7.68) | 0.96  (0.63, 1.29) | <0.001 | 12.34  (12.04, 12.64) | 9.33  (8.90, 9.76) | -3.01  (-3.55, -2.48) | <0.001 | -3.97  (-4.60, -3.35) |  |
| Median | 5 | 6 | - | - | 11 | 8 | - | - |  |  |
| Variance | 24.27 | 35.08 | - | - | 51.79 | 44.94 | - | - |  |  |
| % Abnormal | 4.66%  (3.72, 5.83) | 8.75%  (7.70, 9.94) | 4.09  (2.87, 5.31) | <0.001 | 28.45%  (26.60, 30.37) | 15.80%  (13.57, 18.31) | -12.65%  (-15.65, -9.65) | <0.001 |  |  |
| **Time x sex** | - | - | -0.29  (-0.74, 0.017) | 0.22 | - | - | 0.18  (-0.55, 0.91) | 0.63 | - | - |
| **Country x sex x time** | - | - | - | - | - | - | - | - | 0.47  (-0.40, 1.35) | 0.29 |
| **Emotional problems** | | | | | | | | | | |
| **Males** | | | | | | | | | | |
| Mean | 1.15  (1.07, 1.23) | 1.60  (1.52, 1.68) | 0.45  (0.34, 0.57) | <0.001 | 3.47  (3.36, 3.58) | 2.78  (2.63, 2.93) | -0.69  (-0.88,-0.50) | <0.001 | -1.14  (-1.36, -0.93) | <0.001 |
| Median | 1 | 1 | - | - | 3 | 2 | - | - |  |  |
| Variance | 2.51 | 4.04 | - | - | 6.57 | 5.75 | - | - |  |  |
| % Abnormal | 4.75%  (3.76, 5.97) | 10.27%  (9.15, 11.52) | 5.52%  (4.23, 6.83) | <0.001 | 31.16%  (29.23, 33.17) | 22.86%  (20.32, 25.60) | -8.30%  (-11.58, -5.03) | - |  |  |
| **Females** | | | | | | | | | | |
| Mean | 1.90  (1.80, 1.99) | 2.55  (2.46, 2.65) | 0.66  (0.52, 0.79) | <0.001 | 4.38  (4.27, 4.50) | 3.65  (3.48, 3.82) | -0.73  (-0.94, -0.52) | <0.001 | -1.39  (-1,63, -1.14) | <0.001 |
| Median | 1 | 2 | - | - | 4 | 3 | - | - |  |  |
| Variance | 4.04 | 6.01 | - | - | 7.45 | 7.16 | - | - |  |  |
| % Abnormal | 11.66%  (10.18, 13.31) | 20.67%  (19.14, 22.29) | 9.01%  (7.22, 10.81) | <0.001 | 44.52%  (42.45, 46.60) | 33.24%  (30.28, 36.35) | -11.26%  (-14.94, -7.62) | <0.001 |  |  |
| **Time x sex** | - | - | 0.19  (0.02, 0.36) | <0.05 | - | - | -0.04  (-0.32, 0.28) | 0.78 | - | - |
| **Country x sex x time** | - | - | - | - | - | - | - | - | -0.24  (-0.57, 0.09) | 0.15 |
| **Conduct problems** | | | | | | | | | | |
| **Males** | | | | | | | | | | |
| Mean | 1.05  (0.97, 1.12) | 1.23  (1.16, 1.29) | 0.18  (0.09, 0.27) | <0.001 | 2.17  (2.08, 2.27) | 1.32  (1.21, 1.43) | -0.85  (-1.01, -0.70) | <0.001 | -1.03  (-1.20, -0.86) | <0.001 |
| Median | 1 | 1 | - | - | 2 | 1 | - | - |  |  |
| Variance | 2.04 | 2.50 | - | - | 4.86 | 2.88 | - | - |  |  |
| % Abnormal | 5.99%  (4.82, 7.41) | 8.65%  (7.60, 9.82) | 2.66%  (1.33, 3.97) | <0.001 | 24.11%  (22.35, 25.99) | 11.00%  (9.16, 13.14) | -13.11%  (-15.79, -10.46) | <0.001 |  |  |
| **Females** | | | | | | | | | |  |
| Mean | 1.12  (1.05, 1.19) | 1.07  (1.02, 1.13) | -0.05  (-0.14, 0.04) | 0.29 | 2.39  (2.29, 2.49) | 1.54  (1.41, 1.66) | -0.85  (-1.02, -0.69) | <0.001 | -0.80  (-0.99, -0.63) | <0.001 |
| Median | 1 | 1 |  | - | 2 | 1 | - | - |  |  |
| Variance | 2.00 | 1.92 |  | - | 5.35 | 3.65 | - | - |  |  |
| % Abnormal | 6.62%  (5.46, 8.01) | 6.09%  (5.22, 7.09) | -0.53%  (-1.77, 0.06) | 0.39 | 29.32%  (27.46, 31.26) | 15.40%  (13.19, 17.91) | -13.92%  (-16.93, -10.91) | <0.001 |  |  |
| **Time x sex** | - | - | -0.23  (-0.35, -0.10) | <0.001 | - | - | 0.00  (-0.21, 0.21) | 0.99 | - | - |
| **Country x sex x time** | - | - | - | - | - | - | - | - | 0.23  (-0.02, 0.48) | 0.08 |
| **Hyperactive problems** | | | | | | | | | | |
| **Males** | | | | | | | | | | |
| Mean | 2.94  (2.83, 3.05) | 2.87  (2.77, 2.96) | -0.07  (-0.22, 0.07) | 0.33 | 4.21  (4.08, 4.35) | 2.89  (2.72, 3.07) | -1.32  (-1.55, -1.09) | <0.001 | -1.25  (-1.51, -0.98) | <0.001 |
| Median | 3 | 2 | - | - | 4 | 2 | - | - |  |  |
| Variance | 5.07 | 6.00 | - | - | 9.89 | 7.99 | - | - |  |  |
| % Abnormal | 8.47%  (7.12, 10.1) | 9.24%  (8.15, 10.46) | 0.77%  (-0.07, 0.22) | 0.31 | 25.7%  (23.9, 27.6) | 13.0%  (11.0, 15.3) | -12.7%  (-9.99, 15.5) | <0.001 |  |  |
| **Females** | | | | | | | | | | |
| Mean | 2.43  (2.33, 2.53) | 2.10  (2.02, 2.18) | -0.33  (-0.45, -0.20) | <0.001 | 3.40  (3.28, 3.53) | 2.36  (2.20, 2.52) | -1.04  (-1.25, -0.83) | <0.001 | -0.71  (-0.95, -0.48) | <0.001 |
| Median | 2 | 2 | - | - | 3 | 2 | - | - |  |  |
| Variance | 4.14 | 4.21 | - | - | 8.49 | 6.21 | - | - |  |  |
| % Abnormal | 4.22%  (3.31, 5.36) | 3.55%  (2.92, 4.30) | -0.67%  (-0.16, 0.03) | 0.19 | 15.93%  (14.46, 17.52) | 8.77%  (7.09, 10.80) | -7.16%  (-9.53, -4.80) | <0.001 |  |  |
| **Time x sex** | - | - | -0.25  (-0.43, -0.06) | <0.01 | - | - | 0.28  (-0.02, 0.58) | 0.07 | - | - |
| **Country x sex x time** | - | - | - | - | - | - | - | - | 0.53  (0.18, 0.89) | <0.001 |
| **Peer problems** | | | | | | | | | | |
| **Males** | | | | | | | | | | |
| Mean | 1.22  (1.15, 1.30) | 1.84  (1.77, 1.92) | 0.62  (0.52, 0.72) | <0.001 | 2.01  (1.93, 2.09) | 1.68  (1.56, 1.79) | -0.33  (-0.47, -0.19) | <0.001 | -0.95  (-1.12, -0.77) | <0.001 |
| Median | 1 | 1 | - | - | 2 | 1 | - | - |  |  |
| Variance | 2.28 | 3.62 | - | - | 3.59 | 3.36 | - | - |  |  |
| % Abnormal | 8.13%  (6.92, 9.54) | 17.44%  (16.02, 18.97) | 9.31%  (7.66, 10.96) | <0.001 | 23.90%  (22.14, 25.77) | 16.20%  (14.01, 18.67) | -7.70%  (-10.64, -4.78) | <0.001 |  |  |
| **Females** | | | | | | | | | | |
| Mean | 1.08  (1.01, 1.16) | 1.73  (1.66, 1.80) | 0.64  (0.54, 0.74) | <0.001 | 2.16  (2.08, 2.25) | 1.77  (1.66, 1.89) | -0.39  (-0.54, -0.24) | <0.001 | -1.03  (-1.21, -0.86) | <0.001 |
| Median | 1 | 1 | - | - | 2 | 1 | - | - |  |  |
| Variance | 2.14 | 3.27 | - | - | 4.08 | 3.37 | - | - |  |  |
| % Abnormal | 6.74%  (5.61, 8.08) | 15.64%  (14.28, 17.10) | 8.90%  (7.38, 10.40) | <0.001 | 27.59%  (25.76, 29.49) | 18.0%  (15.64, 20.61) | -9.59%  (-12.68, -6.51) | <0.001 | - | - |
| **Time x sex** | - | - | 0.02  (-0.11, 0.17) | 0.65 | - | - | -0.06  (-0.26, 0.14) | 0.56 | - | - |
| **Country x sex x time** | - | - | - | - | - | - | - | - | -0.08  (-0.33, 0.16) | 0.51 |
| Note: ^Ϯ^ Changes refer to mean differences between continuous scores that were compared over time within each country using t-tests, and across country and time using an interaction between country and time. The percentage of abnormal scores were binary outcomes that were compared within each country using proportions tests. Estimates from ALSPAC use entropy balanced weights that were added as an interaction with the inverse probability weights. For the MCS, sample design weights were added as an interaction with the inverse probability weights. For both Pelotas cohorts, inverse probability weights were used. | | | | | | | | | | |


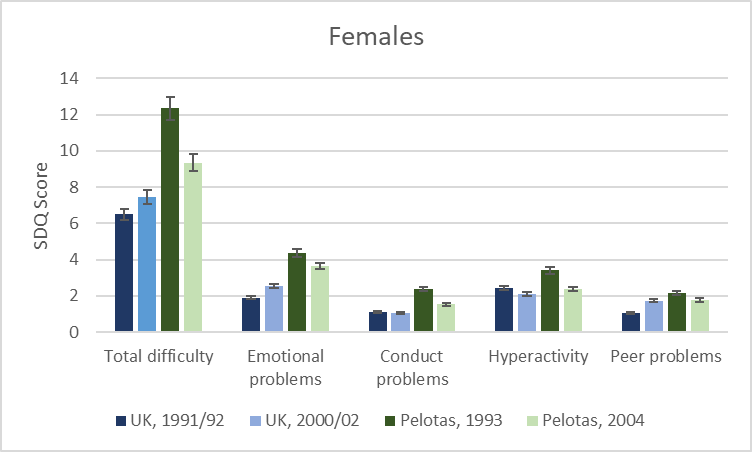

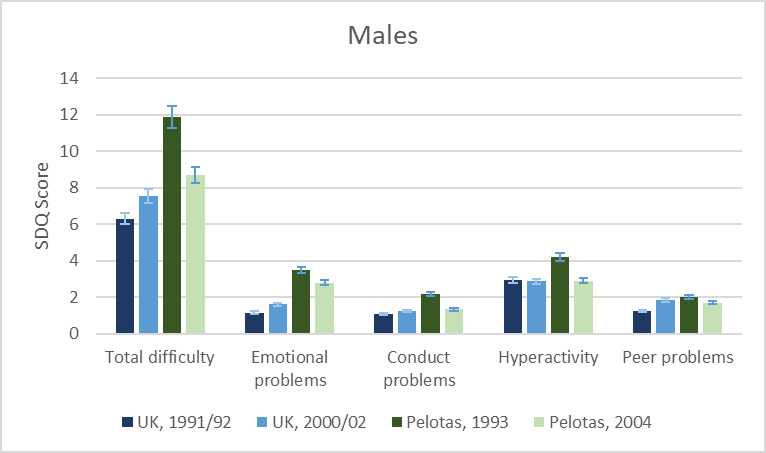


**Supplementary Figure 1:** Change in SDQ total difficulty and subscale scores at age 15/17 among males and females in the UK and Pelotas cohorts. Note total difficulty scores range from 0-40, and individual subscales from 0-10.
